# Exploring late Paleolithic and Mesolithic diet in the Eastern Alpine region of Italy through multiple proxies

**DOI:** 10.1002/ajpa.24128

**Published:** 2020-09-11

**Authors:** Gregorio Oxilia, Eugenio Bortolini, Federica Badino, Federico Bernardini, Valentina Gazzoni, Federico Lugli, Matteo Romandini, Anita Radini, Gabriele Terlato, Giulia Marciani, Sara Silvestrini, Jessica C. Menghi Sartorio, Ursula Thun Hohenstein, Luca Fiorenza, Ottmar Kullmer, Claudio Tuniz, Jacopo Moggi Cecchi, Sahra Talamo, Federica Fontana, Marco Peresani, Stefano Benazzi, Emanuela Cristiani

**Affiliations:** ^1^ Department of Cultural Heritage University of Bologna Ravenna Italy; ^2^ DANTE Diet and Ancient Technology Laboratory Department of Oral and Maxillo Facial Sciences Sapienza University Rome Italy; ^3^ C.N.R. ‐ Istituto di Geologia Ambientale e Geoingegneria Milan Italy; ^4^ Centro Fermi, Museo Storico della Fisica e Centro di Studi e Ricerche Enrico Fermi Rome Italy; ^5^ Multidisciplinary Laboratory The Abdus Salam International Centre for Theoretical Physics Trieste Italy; ^6^ Department of Humanities—Section of Prehistoric and Anthropological Sciences University of Ferrara Ferrara Italy; ^7^ Department of Chemical and Geological Sciences University of Modena and Reggio Emilia Modena Italy; ^8^ Monash Biomedicine Discovery Institute, Department of Anatomy and Developmental Biology Monash University Melbourne Victoria Australia; ^9^ Earth Sciences University of New England Armidale New South Wales Australia; ^10^ Senckenberg Research Institute and Natural History Museum Frankfurt Frankfurt am Main Germany; ^11^ Department of Paleobiology and Environment, Institute of Ecology, Evolution, and Diversity Johann Wolfgang Goethe University Frankfurt Germany; ^12^ Centre for Archaeological Science University of Wollongong Wollongong New South Wales Australia; ^13^ Department of Biology University of Florence Florence Italy; ^14^ Department of Human Evolution Max Planck Institute for Evolutionary Anthropology Leipzig Germany; ^15^ Department of Chemistry “G. Ciamician” University of Bologna Bologna Italy

**Keywords:** dental calculus, Eastern Alpine region, Late Paleolithic, macrowear, Mesolithic, palaeonutrition, stable isotopes

## Abstract

**Objectives:**

The analysis of prehistoric human dietary habits is key for understanding the effects of paleoenvironmental changes on the evolution of cultural and social human behaviors. In this study, we compare results from zooarchaeological, stable isotope and dental calculus analyses as well as lower second molar macrowear patterns to gain a broader understanding of the diet of three individuals who lived between the end of the Late Pleistocene and the Early Holocene (ca., 17–8 ky cal BP) in the Eastern Alpine region of Italy.

**Materials and methods:**

We analyze individuals buried at the sites of Riparo Tagliente (Verona), Riparo Villabruna, and Mondeval de Sora (Belluno). The three burials provide a unique dataset for diachronically exploring the influence of climatic changes on human subsistence strategies.

**Results:**

Isotopic results indicate that all individuals likely relied on both terrestrial and freshwater animal proteins. Even though dental calculus analysis was, in part, hindered by the amount of mineral deposit available on the teeth, tooth macrowear study suggests that the dietary habits of the individuals included plant foods. Moreover, differences in macrowear patterns of lower second molars have been documented between Neanderthals and modern humans in the present sample, due to a prevalence of Buccal wear among the former as opposed to higher values of Lingual wear in modern human teeth.

**Discussion:**

Isotopic analyses have emphasized the contribution of animal proteins in the diet of the three foragers from the Eastern Alpine region. The possible intake of carbohydrate‐rich plant foods, suggested by the retrieval of plant remains in dental calculus, is supported by the signal of macrowear analysis. Moreover, the latter method indicates that the distribution of macrowear in lower second molars (M_2_s) allows us to discriminate between Neanderthals and modern humans within the present reference sample. Overall, our results show these three prehistoric hunter‐gatherers were well adapted to the environment in which they lived exploiting many natural resources.

## 1 INTRODUCTION

Reconstructing prehistoric human diet has always been a key goal of the research into past human behavior. Different proxies and methods have been integrated to obtain a comprehensive overview of prehistoric human dietary strategies and for understanding cultural responses to climate and environmental constraints: isotopic analysis (e.g., Drucker & Bocherens, 2004; Lugli et al., 2019, Wißing et al., 2019); lithic and osseous technology (e.g., Arrighi et al., 2019; Caricola et al., 2018; Collina et al., 2020; Marciani et al., 2019; Semenov, 1964; Stout, 2011); faunal remains (i.e., quantification of faunal remains and analysis of bone surface modifications; Gaudzinski‐Windheuser & Kindler, 2012); and dental remains (i.e., formal assessment of paramasticatory and masticatory activities; Arnaud et al., 2016; Been et al., 2017; Fiorenza et al., 2015; Fiorenza, Benazzi, & Kullmer, 2011; Margherita et al., 2016; Margherita et al., 2017; Oxilia et al., 2015; Oxilia et al., 2017; Riga et al., 2018). In particular, dental wear, dental pathologies and, when preserved, prehistoric dental calculus have shown to be pivotal in obtaining data on diet, cultural habits, and health status (Cristiani et al., 2018; El Zaatari & Hublin, 2014; Fiorenza, 2015; Fiorenza, Benazzi, Oxilia, & Kullmer, 2018; Fiorenza & Kullmer, 2013, 2015; Grippo, Simring, & Schreiner, 2004; Henry, Hudson, & Piperno, 2009; Lussi, 2006; Metcalf, Ursell, & Knight, 2014; Molnar & Molnar, 1990; Oxilia et al., 2018; Radini, Buckley, Nikita, Copeland, & Hardy, 2017; Sameera, Singh, & Nitya, 2017; Sorrentino et al., 2018; Warinner et al., 2014; Weyrich et al., 2017). While successfully applied to the analysis of historical contexts (e.g., Baldoni et al., 2018; Figus et al., 2017; Gismondi et al., 2020; Radini, Nikita, & Shillito, 2016; Vazzana et al., 2018), the proxies mentioned above have sporadically been combined in prehistoric reconstructions (Fiorenza et al., 2015), potentially leading to a lack of information about ancient dietary habits.

Investigating various strands of evidence can be particularly relevant in the case of individuals who lived in periods of climatic changes, which can trigger specific patterns of human‐environment interaction in distinctive regional settings. Between the end of the Last Glacial Maximum (LGM—⁓16.5 ka cal yr BP; Lambeck, Rouby, Purcell, Sun, & Sambridge, 2014) and the early Holocene (after 11.7 ka cal yr BP), climatic temperature oscillations set in motion significant changes in human culture, mobility, adaptive strategies, and population structure across continents.

After the LGM, the climate amelioration triggered a general withdrawal of the main glaciers in the Eastern Alpine region. The retreat of glaciers in the region brought to the repopulation of mountainous areas by animals and human groups (Bertola et al., 2007). The abundant archeo‐osteological evidence and the long‐lasting tradition of prehistoric studies in this area make the Eastern Alpine region significant for exploring the influence of climatic changes on subsistence strategies.

**FIGURE 1:**
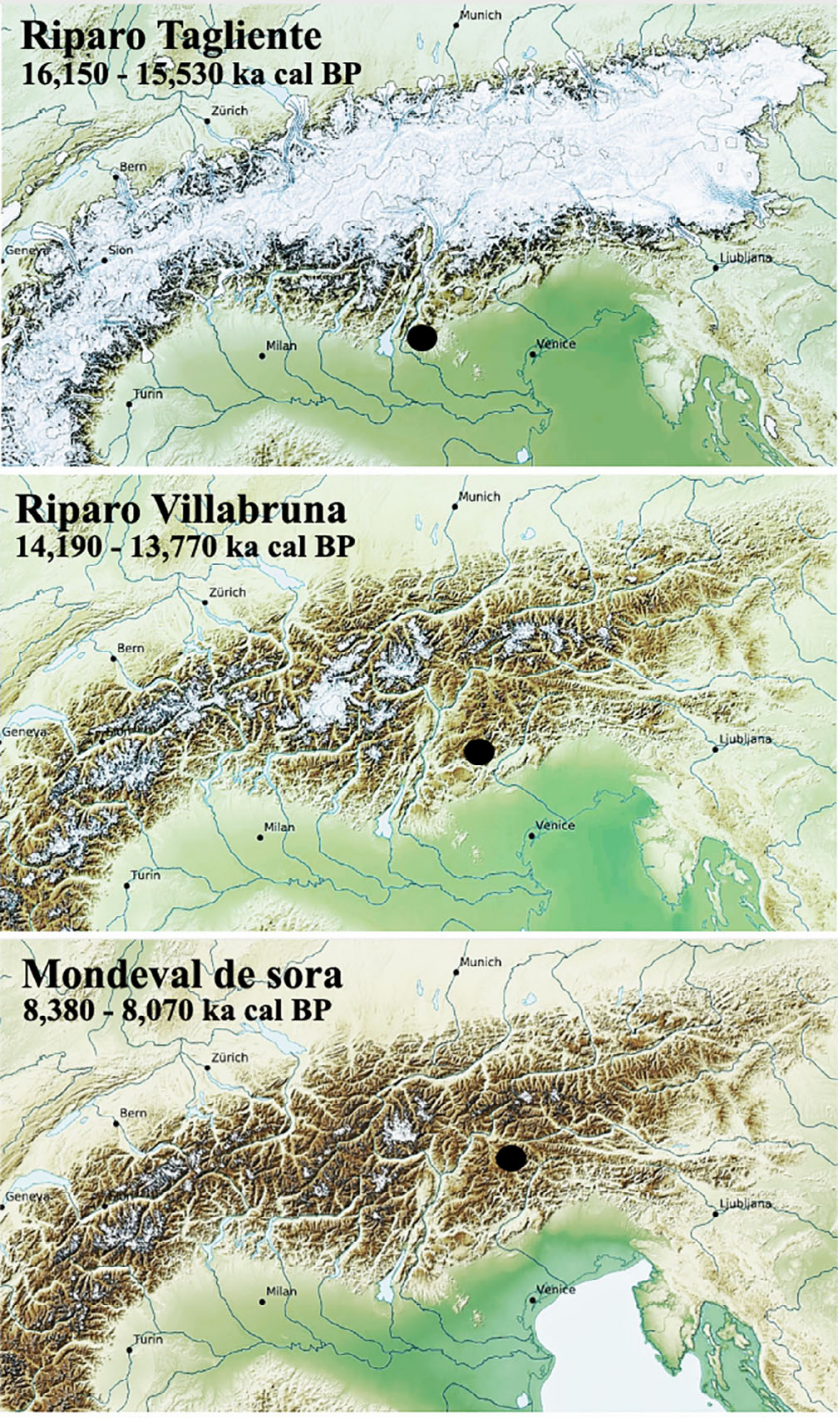
Paleogeographic context of the three individuals buried at the sites of Riparo Tagliente (Verona), Riparo Villabruna and Modeval de Sora (Belluno). Snapshots showing Alpine glaciers extension modeled during three different time intervals. Frames modified from Seguinot et al., 2018 (Modeling last glacial cycle ice dynamics in the Alps: https://doi.org/10.5446/35164, fixed frame version)

In this article, we focus on three foragers buried at Riparo Tagliente (Verona), Riparo Villabruna, and Mondeval de Sora (Belluno). These sites span the period from ⁓16.5 to 8 ka cal BP which falls in the Late Upper Paleolithic and the end of the Late Mesolithic (Figure 1) and are crucial for understanding modalities of human‐environment interaction after the LGM in the analyzed region. To explore prehistoric dietary habits, we first provide the paleoenvironental background of the area between the end of the Late Pleistocene and the early Holocene. Then we combine data obtained through different analytical methods, generally performed independently, such as (a) analysis of faunal assemblages recovered in layers that are coeval to the burials; (b) stable isotope analysis; (c) dental macrowear analysis. Also, we analyzed dental calculus, limited by a small amount of mineralized biofilm preserved on the three individuals, for its potential to inform about dietary strategies. Last, we discuss dietary strategies of the three foragers who lived in the Eastern Alpine region against paleoenvironmental data already available for the region of interest.

### 1.1 Paleoenvironmental and archeological background

The Eastern Alpine region of Italy is one of the best‐suited areas to investigate the environmental and cultural influence on the subsistence and dietary preferences of hunter‐gatherer groups of the last deglaciation. This region is located north of the Po valley, and the Friulian‐Venetian plains in a wide pre‐Alpine belt of mountains peaking over 2000 m, karst plateau elevated to 1,000–1,200 m, dissected by gorges, river valleys, and alpine lakes. During the LGM, European Alps were extensively covered by an ice‐dome reaching a maximum ice extent around ca., 25 ka cal yr BP, when massive Piedmont glaciers advanced onto the Alpine foreland (Ivy‐Ochs et al., 2008; Monegato, Scardia, Hajdas, Rizzini, & Piccin, 2017) and sea‐level dropped of ⁓120 or 149 m (Pellegrini et al., 2015; Maselli et al., 2014). The pre‐Alpine area underwent glacial and peri‐glacial conditions with profound modifications of the ecosystems, the evolution of the physiographic outline of the valley floors (Rossato, 2013), extinction of the less adapted large herbivore and carnivore species (Sala, 1990; Terlato et al., 2018), setting the grounds for the development of the recent ecosystems.

The subsequent Late Glacial period (i.e., the interval between the end of the LGM and the beginning of the Holocene) was punctuated by a series of millennial‐scale climate oscillations and dynamic glacial fluctuations (Seguinot et al., 2018).

After the major glaciers retreated, the evolution of the valley floor was controlled by slope‐damming processes. Larch, scots pine, and mountain pine forests were limited to the foreland and the external belt of Pre‐Alps (Ravazzi et al., 2014; Vescovi et al., 2007). During this period, groups of foragers moved on the southern slope of the Pre‐Alps.

The oldest known archeological site on the Southern slope of the Alps to be re‐occupied by human groups after the LGM was Riparo Tagliente (Fontana et al., 2008, Fontana, Cilli, et al., 2009; Fontana et al., 2018; Naudinot, Tomasso, Tozzi, & Peresani, 2014). The site, located at an altitude of 250 m a.s.l. along the corridor of the Valpantena valley giving access to the Lessini Plateau. A 4.5 m deep stratigraphic sequence, formed by two main deposits separated by a river escarpment, documents lower strata referred to MIS 4–3 occupation with Mousterian and Aurignacian assemblages and upper ones dated to the Late Glacial, attesting a Late Epigravettian occupation. According to radiocarbon dates (calibrated using IntCal09), which range from 17,219–16,687 cal BP (LTL4441A; layer 13a alpha) to 14,572–13,430 cal BP (R‐371; layers 10–8), the Epigravettian series of Riparo Tagliente is one of the most complete in Northern Italy spanning the first part of the Late Glacial until the beginning of the Bølling–Allerød interstadial (Fontana, Cilli, et al., 2009; Fontana et al., 2018). During the first part of the Late Glacial, an individual was buried in the area protected by the overhang of the shelter (Level 16–13; Tagliente 1). A pit opened in historical times partially destroyed the burial and removed the entire skeleton above the pelvis (Table 1). A hemi‐mandible (Tagliente 2) was found within disturbed deposits just outside the rock‐shelter. These deposits probably derived from the partially destroyed Late Upper Paleolithic sequence of the inner area of the shelter, something also suggested by the association of the mandible with Epigravettian archeological materials (Corrain, 1966).

**TABLE 1:** Radiocarbon dates of the burials discovered at the Riparo Tagliente, Riparo Villabruna and Mondeval de Sora.

Sample	AMS	^14^C Age	Err 1σ	Cal BP 68.2%	Cal BP 95.4%	References
Tagliente 1	OxA‐10672	13,190	90	16,010‐15,710	16,150–15,530	Gazzoni et al. 2013
Villabruna	KIA‐27004	12,140	70	14,130‐13,860	14,190–13,770	Vercellotti et al 2008
Mondeval	OxA‐7468	7,425	55	8320‐8190	8,380–8,070	Bronk Ramsey et al. 2002

*Note:* Dates were calibrated in OxCal v.4.3 (Bronk Ramsey 2017) using the IntCal13 calibration curve (Reimer et al. 2013).

The climatic warming at the onset of the Bølling–Allerød (B–A) interstadial, dated ca. 14.7 ka cal BP, promoted the development of alpine habitats to higher altitudes. Such favorable climatic conditions promoted the increase in the number of Epigravettian sites and gradual colonization of high altitudes (i.e., above 1,000 m a.s.l.). The geographic distribution of the settlements is attested between the valley‐bottom areas and the middle mountain range mostly in the vicinity of peat bogs or landmarks, also in the inner Alps and the Dolomitic region (Avigliano, Di Anastasio, Improta, Peresani, & Ravazzi, 2000; Bertola et al., 2007; Broglio & Impronta, 1994–1995; Montoya, Duches, Fontana, Peresani, & Visentin, 2018) and karst plateaux up to an altitude of 1,600 m a.s.l. (Bertola et al., 2007; Broglio, 2001; Broglio & Impronta, 1994–1995; Fiore & Tagliacozzo, 2005; Montoya et al., 2018; Peresani, Bertola, De Stefani, & Di Anastasio, 1999).

Within this context, seasonal settlement systems based on the interplay of sites with complementary functionality developed. Such behavior is well attested at Riparo Tagliente as well as Riparo Villabruna (Aimar et al., 1992; Fontana, Cilli, et al., 2009; Fontana et al., 2018; Naudinot et al., 2014). The latter site, also object of investigation in this article, is located at 500 m a.s.l. in the Venetian Dolomites at the confluence of the Cismòn Valley with Rosna creek and characterized by a group of rock shelters situated in the proximity of the valley bottom (Broglio & Villabruna, 1991). Two meters of deposit have excavated at the site, and an Epigravettian burial was brought to light at the bottom of the stratigraphic sequence. Grave goods, represented by isolated blades, a bone point, a backed knife, a flint blade, a flint core, a stone retoucher and a ball of probable resin and wax, were positioned probably in a bag, on the left forearm near the pelvis (Broglio & Villabruna, 1991). Natural stones and large painted stones collected on the stream bed close to the shelter were used to cover the body after the internment. Radiocarbon dates obtained on a charcoal from the grave sediment (Layer 17A; R‐2023:12,040 ± 150 yr BP; Aimar et al., 1992), and the human cranium (KIA‐27004:12.140 ± 70; Vercellotti

et al., 2008) place the burial at 14,190–13,770 B.P.calibrated using IntCal13 in the OxCal 3.0 program (Ramsey et al., 2009; Reimer et al., 2013; Table 1).

Faunal remains indicate that during the summer and autumn, Epigravettian groups used to occupy higher altitudes to exploit the variety of biological resources (Fiore & Tagliacozzo, 2005; Fiore & Tagliacozzo, 2006; Phoca‐Cosmetatou, 2005a, 2005b, 2009). Overall, red deer and ibex were the most frequent herbivores hunted although chamois, roe deer, and wild boar were also common in some faunal assemblages, while aurochs and horse were less targeted. Specialized hunting of marmots was also practiced (Romandini, Peresani, Gurioli, & Sala, 2012).

The successive Younger Dryas (YD) cold event, which started at 12.7 ka cal BP, had strong effects on stands of thermophilus trees formerly expanded in the forelands of the Southern Alps. Treelines shifted 200–300 m downward, whereas steppe and alpine grasslands with xerophytic shrubs expanded. Unfortunately, the available archeological data are not detailed enough to advance an alternative explanation for supposed continuity in settlement dynamics during the YD (Mussi & Peresani, 2011; Naudinot et al., 2014). The YD oscillation preceded a sharp increase of temperature identifying the final transition to the Holocene. At the beginning of these interglacial conditions, within a few centuries, the timberline reached an altitude of about 2,100 m a.s.l. (Oeggl & Wahlmüller, 1994). Afterward, a rapid cooling and moisture increase occurred at ca., 8.2 ka cal BP, which is clearly defined in the Greenland ice core δ^18^O records, favoring the expansion of spruce (*Picea* sp.) and silver fir (*Abies* sp.) in the subalpine belt (Pini, 2002).

During the early Holocene, the whole of north‐eastern Italy was covered by forests. In the Alps, the belt of territory between 1,700 and 2,100 m asl was occupied by Larix–Pinus cembra forests, and at around 10,500 BP, the tree‐line reached 2,250 m (Drescher‐Schneider, 2009; Wick, 1994). This territory was occupied by Mesolithic hunter‐gatherers attested from the present coastal areas of the Northern Adriatic to the inner Alpine highlands. Intense colonization of the central‐eastern Italian Alpine region is documented with evidence of occupation both in the mountains and on valley‐bottoms. We can thus infer a social organization based on small size groups with mobility based on seasonal displacements from the plain/valley‐bottoms to highland territories through the main drainage systems Highland sites were situated at recurrent locations (in the proximity of small lakes and passes, along panoramic crests, either open‐air or under the overhang of erratic boulders) and altitudes, mostly between 1,900 and 2,300 m a.s.l. and occupied during the warmer season of the year (Broglio, 1980; Broglio & Lanzinger, 1990; Fontana & Visentin, 2016; Visentin et al., 2016). During the middle Sauveterrian, for instance, hunting of ungulates was particularly relevant besides other small prey such as pikes and cyprinids, sweet water mollusks, and marsh turtles in the valley bottoms (Wierer and Boscato, 2006; Bazzanella. Betti, & Wierer, 2007).

One important site for understanding the Late Mesolithic occupation of the region is Mondeval de Sora. This site is located at 2,150 m a.s.l. under the overhang of a large erratic boulder on a terrace in the high valley of the Cordevole River (Belluno Dolomites). Traces of human occupation were unearthed under two sides of the boulder (Sectors I and III; Alciati et al., 1994; Fontana & Guerreschi, 2003; Fontana, Guerreschi et al., 2009; Fontana, Govoni, et al., 2009; Valletta, Fontana, Bertola, & Guerreschi, 2016). In Sector I, located along the south‐western side, a complex stratigraphic series was explored over a surface of about 60 m^2^ and yielded layers dated between the Early Mesolithic and Medieval age. Mesolithic layers were preserved only in the southern portion of the site. The Early Mesolithic (Sauveterrian) sequence documents some dwelling structures associated to two main layers containing a rich lithic assemblage along with faunal and charcoals remains (Alciati et al., 1994; Berto, Luzi, Guerreschi, Fontana, & Valletta, 2016; Colombo et al., 2016; Fontana, Govoni, et al., 2009; Fontana & Guerreschi, 2003; Fontana, Guerreschi, et al., 2009; Fontana & Vullo, 2000; Thun Hohenstein et al., 2016; Valletta et al., 2016). The Late Mesolithic (Castelnovian) layers, although disturbed by the later occupation of the site, yielded a well‐preserved burial of a young individual. The skeleton lied in a supine position within a pit naturally delimited by two dolomite boulders with the lower part of the body covered with stones (Fontana, 2006; Fontana et al., 2016; Gerhardinger & Guerreschi, 2006). The individual was accompanied by a rich repertoire of burial goods consisting of 60 items of various typologies laid around the body, and on the right side of the skeleton, a small patch of red ochre was recovered. One direct AMS radiocarbon date on the skeleton yielded a result of 7,425 ± 55 years BP (OxA‐7468) (8,380–8,070 cal BP; Fontana, Guerreschi, Bertola, Briois, & Ziggiotti, 2016) calibrated using IntCal13 in the OxCal 3.0 program (Ramsey et al., 2009; Reimer et al., 2013; Table 1).

Eventually, the ecological shift documented during the Late Upper Paleolithic and Mesolithic is reflected in the diversification of hunting technology and the exploitation of both aquatic and terrestrial resources (Gazzoni et al., 2013; Gazzoni et al., under review; Mannino, Di Salvo, et al., 2011; Mannino, Thomas, et al., 2011) besides megafauna hunting (Germonpré, Sablin, Khlopachev, & Grigorieva, 2008).

## 2 MATERIALS AND METHODS

### 2.1 Riparo Tagliente

Two different human remains have been discovered: Tagliente 1 is a burial preserved from the pelvis to the feet and belonging to a ca. 20–29‐year‐old young adult male (Bartolomei et al., 1974; Corrain, 1977), Tagliente 2, consists of a hemi‐mandible with teeth in sockets (Figure S1).

Considering the uncertainty of the stratigraphic position of Tagliente 2, we believe these two human remains likely belonged to different individuals. In this study, we focus our attention on Tagliente 2 to implement morphometric and morphological information previously published (Corrain, 1966). The teeth have been virtually extracted (Figure S1) and analyzed (Figure S2) in a digital model generated from μCT data. Deposits of dental calculus on the teeth of the hemi‐mandible of Tagliente 2 were removed from the original specimen for conservative cleaning procedures. A minimal quantity of the mineralized biofilm left on the first molar was sampled (ca., 0.4 mg).

### 2.2 Riparo Villabruna

The complete skeleton discovered at Riparo Villabruna belongs to a ca. 25‐year‐old adult male, 167 cm tall. Overall, the human remains are in an excellent state of preservation (Vercellotti et al., 2008). However, the distal portions of both lower limbs are incomplete due to damage that occurred during the work that led to the discovery of the site. Both the cranium and the mandible are complete, although the lower left first incisor has been lost postmortem, and the lower‐left second premolar as well as the lower right second molar were not preserved. Dental calculus from the Villabruna skeleton was removed for conservative cleaning procedures. Only a minimal quantity of dental calculus was left and sampled for the analysis (ca., 0.5 mg).

### 2.3 Mondeval de Sora

The buried individual from Mondeval de Sora belongs to a ca. 40‐year‐old male, 165 cm tall. The remains are incredibly well preserved, lacking only the most fragile components of the facial part and some distal bones of the left foot. The preservation of the skeleton allowed various studies to be performed, including stable isotope analysis (Gazzoni, 2011). Dental calculus was removed for conservative cleaning procedures, and the very small sample left was collected for micro‐debris analysis (ca., 0.8 mg).

### 2.4 Comparative sample for the macrowear analysis

The sample used in this study is conservative and consists of second mandibular molars (M_2_) of hunter‐gatherers. The specimens listed in Table S1 include 33 fossil individuals from European and Levant Middle/Upper Paleolithic (i.e., Neanderthals and anatomically modern humans) and Epipaleolithic Natufian individuals. Each individual was assigned to an ecogeographic group based on literature (Fiorenza, 2015), to geographic areas based on the region of discovery, to climatic classes based on the relevant Marine Isotope Stage (MIS), and to the respective species (*Homo neanderthalensis* and *Homo sapiens*).

In this work, already available data on the analyzed human and faunal remains (e.g., the isotopic analysis and zooarcheological assemblages for Tagliente 1 and Riparo Villabruna) are discussed against the results obtained through the application of different methods (macro‐wear, isotope, and dental calculus analyses) on Tagliente 2, Villabruna and Mondeval individuals.

### 2.5 Data acquisition

As far as Villabruna specimen is concerned, the high resolution of the polygonal models of the M_2_, obtained by μCT images (Oxilia et al., 2015), allowed us to avoid the use of smoothing of the meshes preserving the original surface from the segmented μCT data.

The hemi‐mandible from Riparo Tagliente structures were visualized by high‐resolution μCT at the Multidisciplinary Laboratory of the Abdus Salam International Centre of Theoretical Physics (Trieste, Italy), using a system specifically designed for the study of paleontological and archeological materials (Tuniz et al., 2013). The μCT acquisition of the complete specimen was carried out by using a sealed X‐ray source (Hamamatsu L8121‐03) at a voltage of 110 kV, a current of 90 μA and with a focal spot size of 5 μm. The X‐ray beam was filtered by a 1 mm‐thick aluminum absorber. A set of 1,800 projections of the sample were recorded over a total scan angle of 360° by a flat panel detector (Hamamatsu C7942SK‐25) with an exposure time/projection of 1.5 s. The resulting μCT slices were reconstructed using the commercial software DigiXCT (DIGISENS SAS) in 32‐bit format and obtaining an isotropic voxel size of 41.33 μm.

To avoid any damage, we preferred to acquire macrowear data from a dental cast of the Mondeval de Sora individual. Original dentitions and high‐resolution dental replicas were molded using a low‐viscosity polyvinylsiloxane impression material (President Light‐body; Coltene, Switzerland). The replicas were then produced using special gypsum (EverestH Rock, KaVo), possessing nonreflective properties and optimized for light surface scanning (Fiorenza, Benazzi, & Kullmer, 2009). Three‐dimensional digital models were generated using a high‐resolution (up to 50 μm) Artec Eva‐S Spider 3D blue light technology scanner. Collection and alignment of the scan‐data point clouds were carried out using Artec studio 12 Professional software.

Even though we used different approaches (μCT and surface scan of dental casts) to acquire the original sample, virtual models do not show significant differences in terms of reliability if compared to the original morphology. The 3D virtual models were further processed using Geomagic Design X (3D System Software) in order to remove errors and degenerate/duplicate triangles.

### 2.6 Faunal assemblage analyses

Taxonomic and skeletal identifications were based on the reference collections stored at the Department of Humanities of the University of Ferrara and the Department of Anatomy, Pharmacology and Forensic Medicine of the University of Torino. Taphonomical analysis of faunal assemblages was performed by following protocols previously published (Aimar et al., 1992; Fontana, Cilli, et al., 2009; Thun Hohenstein et al., 2016; see Supporting information). As far as Mondeval de Sora and part of Villabruna assemblages are concerned, we only provided preliminary new data (Table 2).

**TABLE 2:** Composition of the faunal assemblages coeval of Riparo Tagliente (Layer 13. SSUU 409‐410‐416‐417‐418‐420), Riparo Villabruna (layers 16–17), and Mondeval de Sora (Thun Hohenstein et al., 2016; SU 7 + 7II)

	Tagliente layer 13 SSUU 409‐410‐416‐417‐418‐420		Villabruna layers 16–17		Mondeval SU 7 + 7II	
*Taxa*	NISP	%	NISP	%	NISP	%
*Lepus timidus*	1	0.1				
*Lepus* sp.	11	1.0				
*Marmota marmota*	305	28.8				
*Castor fiber*	1	0.1				
Total Lagomorpha and Rodentia	318	30.0				
*Canis lupus*	4	0.4				
*Vulpes vulpes*	11	1.0				
*Ursus arctos*	15	1.4				
*Meles meles*	5	0.5				
*Felis silvestris*	1	0.1				
*Lynx lynx*	1	0.1				
*Panthera leo spelaea*	5	0.5				
cf. *Panthera leo spelaea*	2	0.2				
Total Carnivora	44	4.2				
*Sus scrofa*	17	1.6	2	2.2	3	3.9
*Alces alces*	11	1.0				
cf. *Alces alces*	5	0.5				
*Cervus elaphus*	204	19.3	6	6.5	32	42.1
*Capreolus capreolus*	94	8.9	2	2.2	6	7.9
Cervidae					3	3.9
*Bison priscus*	3	0.3				
*Bos/Bison*	42	4				
*Capra ibex*	307	28.9	52	56.5	5	6.7
*Rupicapra rupicapra*	14	1.3	21	22.8		
Caprinae			2	2.2	27	35.5
Ungulata			7	7.6		
Total Ungulata	697	65.9	92	100	76	100
Total unidentified	115,039		28			
Aves	50					
						
Total NISP	1,109		92		76	
Total NR	116,098		120			

### 2.7 Stable isotopes of Mondeval de Sora and Tagliente 2

Stable isotopes of collagen from Tagliente 2 (from the root of the lower left first molar) were measured using a Thermo Finnigan Flash EA coupled to a Delta V isotope ratio mass spectrometer at the Max Planck Institute for Evolutionary Anthropology, Leipzig (MPI‐EVA) (see Supporting information and Table 3).

**TABLE 3:** Bone samples from Riparo Tagliente (Gazzoni, 2011), Riparo Villabruna (Vercellotti et al., 2008), and Mondeval de Sora (Gazzoni, 2011)

Site	ID sample	Taxonomy	Skeletal portion	%C	%N	C:N	δ^13^C_(PDB‐1)_	δ^15^N_(AIR)_
Tagliente	V_RT_1	*Homo sapiens*	Rib	36.1	12.8	3.3	−18.4	13.0
Tagliente	R‐EVA 1606 R	*Homo sapiens*	M1 root	44.9	15.2	3.4	−19.5	11.5
Tagliente	V_RT_2	*Capra ibex*	Humerus	28.8	11.2	3.0	−18.9	1.2
Tagliente	V_RT_3	*Capra ibex*	Humerus	34.8	12.3	3.3	−19.1	2.5
Tagliente	V_RT_4	*Cervus elaphus*	Metatarsal	34.2	12.7	3.1	−20.0	2.1
Tagliente	V_RT_5	*Cervus elaphus*	Tibia	32.2	11.5	3.2	−20.5	2.0
Tagliente	V_RT_6	*Capreolus capreolus*	Metacarpal	33.5	11.9	3.3	−20.5	5.0
Tagliente	V_RT_7	*Rupicapra rupicapra*	Metatarsal	34.0	12.4	3.2	−18.4	2.7
Tagliente	V_RT_8	*Bos/Bison*	Metacarpal	34.4	12.2	3.3	−19.6	5.8
Tagliente	V_RT_9	*Marmota marmota*	Ulna	31.7	11.2	3.3	−19.8	4.8
Tagliente	V_RT_10	*Marmota marmota*	Radius	31.9	11.2	3.3	−20.6	4.2
Tagliente	V_RT_11	*Vulpes vulpes*	Metatarsal	36.9	13.1	3.3	−19.3	9.4
Tagliente	V_RT_12	*Sus scrofa*	Mandible	34.0	12.0	3.3	−19.4	5.0
Villabruna	S‐EVA‐415	*Homo sapiens*	Fibula	‐	‐	3.6	−19.7	8.0
Villabruna	S‐EVA‐416	*Cervus elaphus*	‐	‐	‐	3.6	−19.8	1.6
Mondeval	V_Mond_1	*Homo sapiens*	Rib	34.3	12.4	3.2	−19.9	9.1
Mondeval	V_Mond_2	*Capra ibex*	Radius	33.3	11.9	3.3	−19.3	1.8
Mondeval	V_Mond_3	*Capra ibex*	Phalanx	33.8	11.8	3.3	−19.2	2.1
Mondeval	V_Mond_4	*Cervus elaphus*	Phalanx	36.2	12.8	3.3	−21.7	4.1
Mondeval	V_Mond_5	*Cervus elaphus*	Tibia	32.7	11.6	3.3	−21.7	3.2
Mondeval	V_Mond_6	*Capra ibex*	Metatarsal	33.4	11.7	3.3	−19.6	1.9

Mondeval bone samples (Table 3) for stable isotope analysis were treated for collagen extraction at the Laboratoire de Biochimie de l'Unité d'Anthropologie de Marseille (France), following protocols previously published (Bocherens et al., 1997; Longin, 1971; see Supporting information). Collagen was then analyzed for stable isotopes at the Iso‐Analytical lab (Sandbach, UK.; Gazzoni, 2011).

### 2.8 Dental calculus analysis

Most of the dental calculus deposits on the teeth of the three skeletons were removed before the time of our analysis for conservation/cleaning purposes. As a consequence, very little mineralized deposit was left and sampled from the Mondeval de Sora individual (0,7 mg), Villabruna and Tagliente 2 (ca., 0,5 mg and 0,4 mg, respectively). Subgingival dental calculus was sampled with sterile disposable blades and saved in sterile centrifuge tubes. Powder‐free gloves were worn at all times during the sampling (see Supporting information). Samples were transported to the DANTE—Diet and Ancient Technology laboratory (Sapienza University) for analyzing masticatory, dietary and environmental microfossils entrapped in their matrix.

Ancient samples were processed in a dedicated clean space and strict procedures were followed to avoid any modern contamination of the sample and the working area (see Supporting information).

Once in the laboratory, calculus samples were put into 1.5 ml sterile tubes, filled with ultrapure water, and agitated using a “vortex” machine to shake any soil particles adhering to their surfaces. The calculus was then removed, washed in ultrapure water and placed again into a sterile centrifuge tube. The sequence was repeated until water washed off clean. Calculus samples were then examined using a stereomicroscope. Any soil still adhering was removed using a fine acupuncture needle dampened with 0.6 N HCl. All the liquids produced during the decontamination were put aside and later analyzed as a control check. The “clean” calculus samples were then inserted in tubes and covered with HCl 6 N. Powder‐free gloves and masks were always used during the procedure. Once dissolved, the calculus was pipetted out and mounted on a glass slide with a drop of 50:50 mixture of glycerol and water. Slides were observed using a Zeiss Imager cross‐polarized transmitted light as well as a NIKON ECLIPSE cross‐polarized microscopes, both equipped with DIC prism and examined at magnifications ranging from ×200 to ×630. Each type of microfossil recovered in dental calculi was described, recorded and photographed. Starch granule identification was achieved using methodologies and criteria known and accepted in the field of modern and ancient starch granules research (Henry et al., 2009; Henry, Brooks, & Piperno, 2011; Yang & Perry, 2013). In addition to this, plant micro‐remains entrapped in dental calculus were interpreted using a modern reference collection of micro‐debris extracted from more than 200 species of plants native to the Mediterranean region stored at the DANTE laboratory and the Department of Archeology at the University of York. Our reference collection also included animal dietary debris (e.g., muscle, skin, fur particles, fish meat, and scales), inorganic nondietary debris that can be trapped in dental calculus during crafting activities (e.g., plant fibers used for cordage, wood particles, etc.), and minerals (e.g., ochre). Our interpretation of bird feather fragments is based on both published literature (Dove, 1998; Dove & Agreda, 2007; Dove & Koch, 2011; Harwood, 2011) and an extensive reference collection including aquatic bird species from the Anatidae, Strigidae, and Accipitridae families. Feather fragments were primarily interpreted based on the node shape and width, node density per mm, and pigmentation.

### 2.9 Dental macrowear analysis

Dental macrowear refers to general lifetime dental tissue loss resulting in macroscopic relief alteration. Several physical and chemical factors are involved in tissue reduction, depending on food choice, environmental setting (El Zaatari & Hublin, 2014; Fiorenza, 2015; Kullmer et al., 2009; Lussi, 2006) and endogenous organismic biology (Grippo et al., 2004; Lussi, 2006). There are also other variables responsible for the specific appearance of dental wear patterns such as asymmetry of the masticatory system (Kimoto et al., 2000; Molnar & Molnar, 1990; Oxilia et al., 2018), bruxism (Sameera et al., 2017) para‐masticatory activities (Fiorenza & Kullmer, 2013, 2015) dental treatment (Oxilia et al., 2015; Oxilia et al., 2017) and food processing methods (Metcalf et al., 2014).

Occlusal wear facet areas (polished enamel areas with well‐defined margins) of M2s were analyzed using the occlusal fingerprint analysis (OFA) method, a digital approach used to reconstruct the major mandibular movements occurring during the rhythmic chewing cycle (Kullmer et al., 2009). Wear facets were manually mapped on each digital surface model, and labeled according to the numbering system created by Maier and Schneck (1981) and later modified by Kullmer et al. (2009).

Wear facets can be produced by occlusal tooth to tooth contact during masticatory processes. The vertical movement (puncture‐crushing) is the first step of the masticatory cycle in mammals (Crompton & Hiiemae, 1970; Hiiemäe & Crompton, 1971; Kay & Hiiemae, 1974) during which the dental wear produces rough surfaces blunting the cusp tips, generated by the contact of the tooth surface with food and any other extrinsic materials (i.e., grit or sand; Kay, 1977; Stone, 1948). Once food has been partitioned, a rhythmic chewing phase (or power stroke) follows where the jaw movements are guided by the occlusal relief of the upper and lower teeth (Crompton & Hiiemae, 1970; Kay & Hiiemae, 1974) insalivating the food particles into a bolus. The power stroke is divided into two phases (Hiiemae & Kay, 1972; Kay & Hiiemae, 1974): the first (Phase I, divided in Lingual and Buccal) happens when opposite molar cusps tend to slide past each other moving to centric occlusion (maximum intercuspation). The second phase (Phase II) is an anterior‐medial movement, where the lower molars move out of occlusion. The chewing cycle terminates with the opening of the jaw.

The relative surface area of the wear facets attributed to Buccal Phase I, Lingual Phase I, and Phase II of the occlusal power stroke was computed by summing the absolute areas (in mm^2^) belonging to the same phase and dividing this sum by the total occlusal wear area. The resulting relative values (proportions) were visualized in a ternary plot, describing the relative proportions that sum to 1 or 100%.

The presence of significant differences in the distribution of each macrowear variable was investigated among groups defined by species, ecogeographic zones, geographic areas, and MIS‐based groups by applying Kruskal–Wallis tests (*α* = .05) and posthoc, two‐tailed Mann–Whitney U tests for independent sample design (*α* = .025). In the case of species, one‐tailed Mann–Whitney tests (*α* = .05) were run based on observation of variable distributions. To avoid problems of multiple testing, all Mann–Whitney *p*‐values were treated with Bonferroni correction. We measured effect size to assess the magnitude of the difference between ecogeographic and MIS‐based groups of modern humans using Cohen's *d* for unpaired samples with pooled standard deviation and Hedge's correction for small sample size via the Cohen's *d* function of the package effsize in R (Torchiano, 2018) The presence of population structure ascribable to the different grouping criteria was assessed by applying Analysis of Molecular Variance (AMOVA; Excoffier, Smouse, & Quattro, 1992) to the pairwise Euclidean distance computed on all reference individuals, in order to use all three macrowear variables at once.

## 3 RESULTS

### 3.1 Faunal assemblage analyses

The large mammal assemblage from the Late Epigravettian series of Riparo Tagliente shows differences in composition between the lower (17–13) and the upper layers (12–5). While the former layers reflect a dominance of species that lived in open environments, the latter ones document a sensible increase of taxa adapted to milder climates, which attest for the diffusion of woodland areas in the region (Fontana, Cilli, et al., 2009). The composition of the small mammal assemblage confirms such a pattern (Berto, Luzi, Montanari Canini, Guerreschi, & Fontana, 2018). The large mammal assemblages, chronologically coherent with the burial event (Layers 16–13 and correlated Stratigraphic Units), are dominated by open environment species, mainly ungulates (Table 2) (Fontana et al., 2018; Gazzoni et al., 2013). Carnivores, lagomorphs and rodents are also well represented, especially *Marmota marmota* (around 30%). As far as freshwater fish is concerned, bones were also present and currently under investigation. Butchery marks consisting of cut marks and scraping marks from skinning, defleshing, disarticulation, and tendon removal on 10% of ungulates (ibex, roe deer, red deer, and chamois) as well as percussion notches for marrow extraction are documented. Several marmot bones exhibit cut marks. Burned fragments are also frequent (Fontana, Cilli, et al., 2009; Rocci Ris, 2006).

The faunal assemblage from the Late Epigravettian occupation layers of Riparo Villabruna shows a prevalence of ungulates mainly represented by *Capra ibex*, *Cervus elaphus*, and *Rupicapra rupicapra* (Aimar et al., 1992; Table 2). The lower layers (17–10) are dominated by Caprinae, suggesting cold and dry climatic conditions. In the upper layers (9–3), the increase of red deer remains suggests a more temperate and wet climate.

As far as the layers of the burial (16 and 17) are concerned, the species mainly hunted were ungulates such as *C. ibex* (56,5%), *R. rupicapra* (22.8%), and *C. elaphus* (6.5%; Table 2). Butchery marks are present on 21% of the total analyzed bones with traces of skinning, defleshing, disarticulation, and removal of tendons. Only six burned fragments have been identified.

The faunal assemblage from layers coeval to the Castelnovian burial of Mondeval de Sora is very small, partially mixed due to postdepositional events. A prevalence of undeterminable fragmentary bones, not useful for paleoenvironmental and paleoeconomic analyses, is also documented. Nonetheless, considering Mesolithic occupation layers in their whole *C. elaphus* is the most represented taxon (Table 2). Bone and antler remains were also found among the grave goods. These include one bone elk point, several red deer bones, and antler artifacts (including one harpoon), seven red deer pierced atrophic canines, one wild boar canine (Alciati, 1992; Fontana et al., 2016).

### 3.2 Stable isotope analysis

**FIGURE 2:**
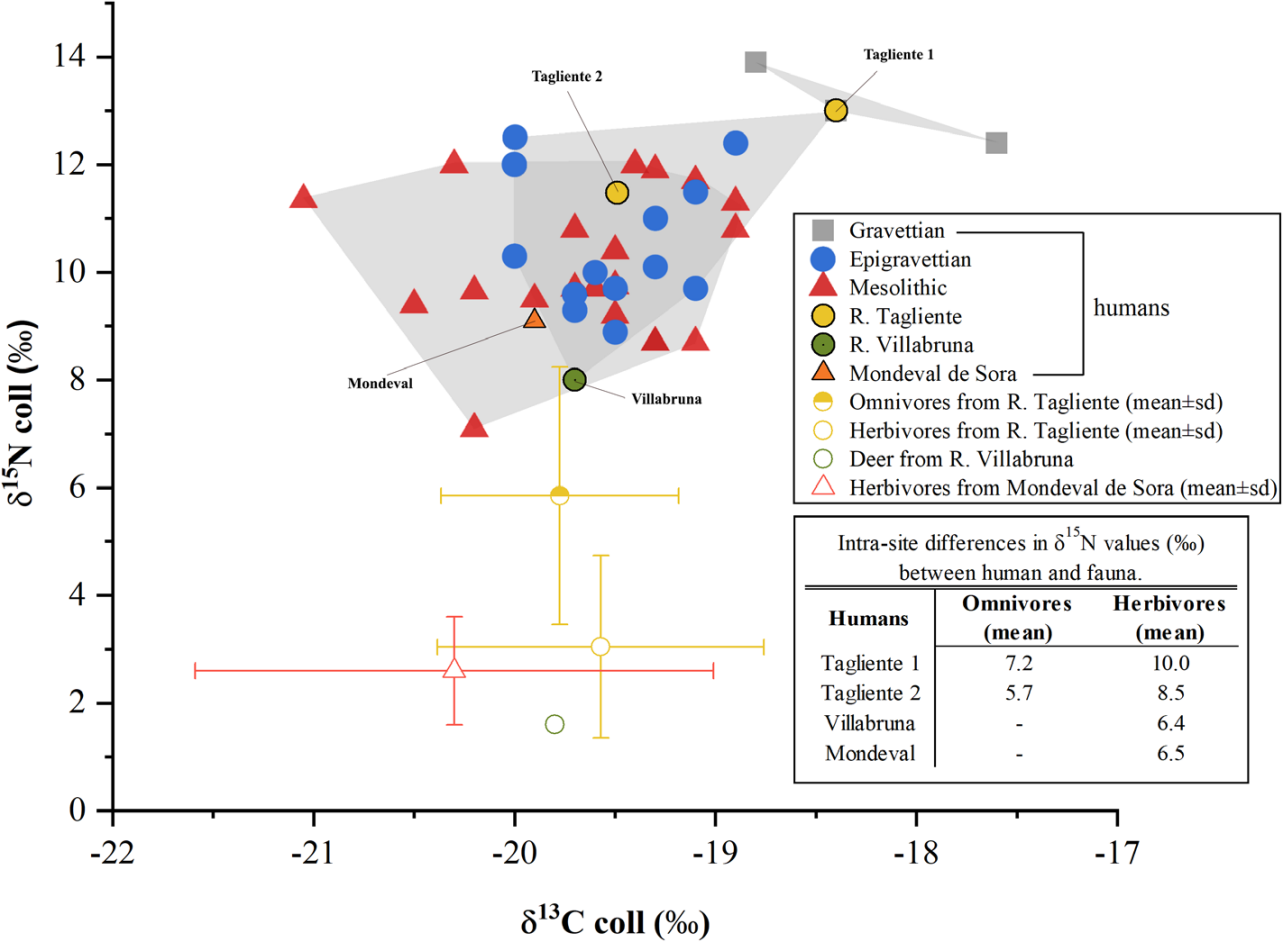
Bone collagen stable isotope values of human remains from Upper Paleolithic (Gravettians are squares in gray; Epigravettians are circles in blue) and Mesolithic (triangles in red) sites in Italy (data from literature; Craig et al., 2010; Di Maida, Mannino, Krause‐Kyora, Jensen, & Talamo, 2019; Floris, Melis, Mussi, Palombo, & Iacumin, 2012; Francalacci & Tarli, 1988; Gazzoni, 2011; Gazzoni et al., 2013; Mannino, Di Salvo, et al. 2011; Mannino, Thomas, Leng, Di Salvo, & Richards, 2011; Mannino et al., 2012; Mannino et al., 2015; Pettitt, Richards, Maggi, & Formicola, 2003; Vercellotti et al., 2008) see Table S2. Faunal data from Riparo Tagliente, Riparo Villabruna and Mondeval de Sora are also shown (see Table 3). In the inset, intrasite differences in δ^15^N between human and (mean) fauna data are summarized

As far as Tagliente 2 is concerned, the value of δ^13^C is −19.5‰ while the δ^15^N is equal to 11.5‰ (Figure 2 and Table 3). Compared with the fauna from the same site, Tagliente 2 δ^13^C values are 0.3 and 0.1‰, respectively higher than omnivore (*M. marmota*, *Vulpes vulpes*, *Sus scrofa*) and herbivore (*C. ibex*, *C. elaphus*, *Capreolus capreolus*, *R. rupicapra*, *Bos/Bison*) mean. At the same time, δ^15^N values are 5.7 and 8.5‰ higher than omnivore and herbivore mean, respectively. The human isotope signature suggests a high trophic level for the Tagliente individual that might indicate a firm reliance on terrestrial animal proteins and possibly fish.

At Mondeval de Sora, the δ^13^C value of human bone collagen is −19.9‰, while the δ^15^N value is 9.1‰ (Figure 2 and Table 3). From the same site, three *C. ibex* samples range between −19.2 and −19.6‰ for carbon (avg: −19.4 ± 0.2‰; 1σ) and between 1.8 and 2.1‰ for nitrogen (avg: 1.9 ± 0.2‰; 1σ), while two *C. elaphus* specimens show a carbon isotope composition of −21.7‰ and a nitrogen isotope composition ranging from 3.2‰ and 4.1‰ (avg: 3.6 ± 0.6‰; 1σ). Human isotopic values suggest a strong reliance on animal proteins (Gazzoni, 2011; Gazzoni et al., under review).

### 3.3 Dental calculus

Due to the minimal amount of the calculus left on the teeth of the analyzed individuals, we can only speculate about the role that plant food held in their diet. Our results should be considered for their potential to inform about the awareness of certain plant species but not as the clear evidence of their regular use by the analyzed foragers.

**FIGURE 3:**
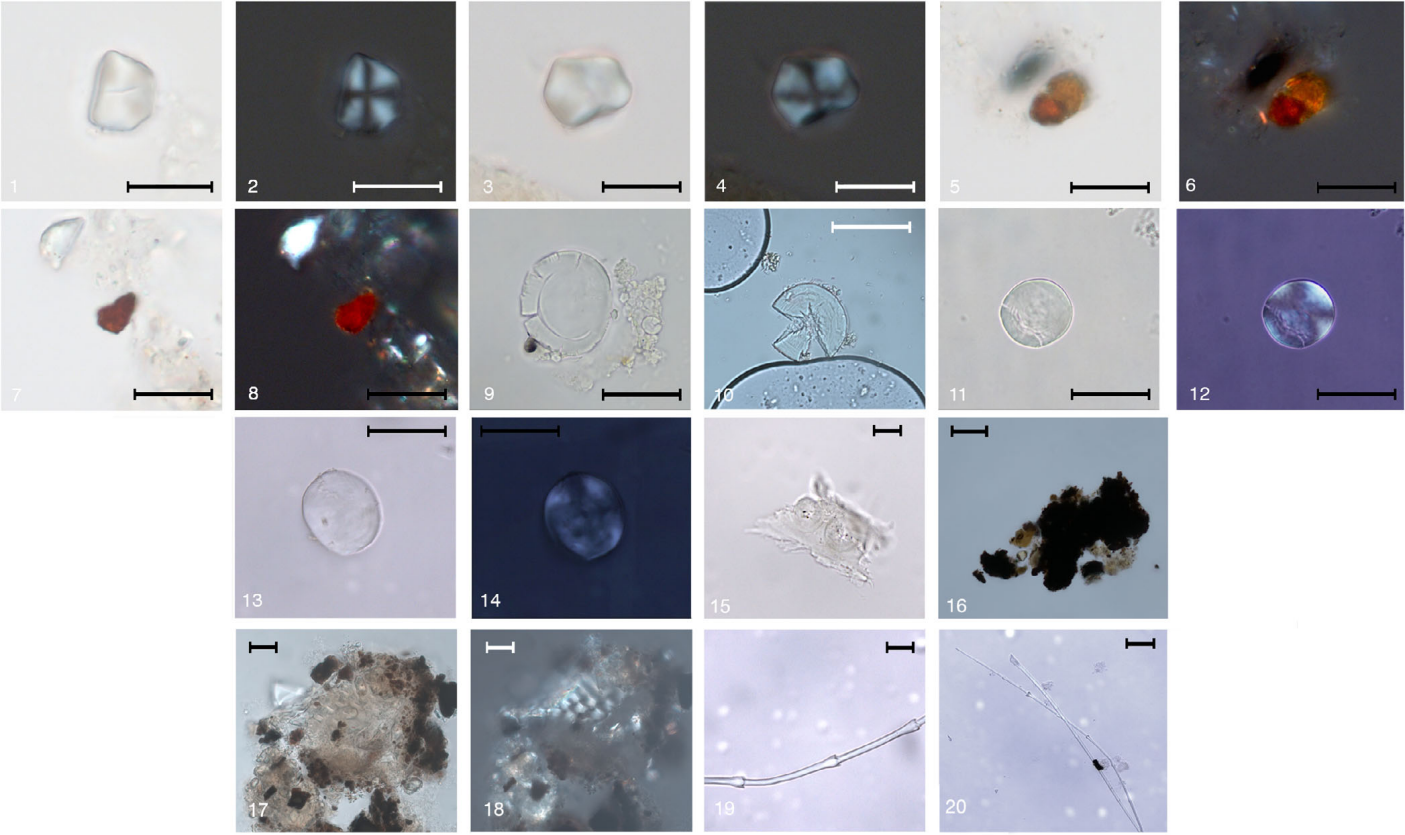
Starch and micro‐debris recovered in dental calculus from Riparo Tagliente, Riparo Villabruna and Mondeval de Sora individuals. (1–4) Small irregular starch granules with central hilum and central fissures from Tagliente; (5–8) mineral debris from Villabruna; micro‐debris from Mondeval de Sora: (9) large sub‐round granule occurring in bimodal distribution; (10–14) large sub‐round granules; (15) egg‐shape aggregate of starch granules; (16) wood particle showing tracheids and bordered pits. The bar is 10 μm; (17) charcoal remains embedded in dental calculus; (18–19) plant structure enclosed in calculus; (20–21) feather particles with evident nodes. The bar is 20 μm. The bar is 10 μm (1–8) and 20 μm (9–21)

In the individual of Riparo Tagliente (Tagliente 2), six irregular polyhedral starch granules (ca.10 μm in length) with central hilum and central fissures were found (Figure 3)1–4. While the interpretation of such irregular grains remains unspecific, our experimental reference collection and published data show that similar granules of around 10 μm in size are found in the Paniceae/Andropogoneae tribe of the Poaceae grass family.

Tagliente 2 displayed very few plant micro remains in the calculus sample, including one small oval starch with central hilum and a few particles of charcoal. Interestingly, plenty of red mineral residues were found entombed in the calculus, the microscopic features of which are consistent with experimental particles of ochre when observed in transmitted polarized light (Figure 35–8).

Plant micro‐remains consistent with starch granules, wood, and other plant fibers as well as micro‐particles of charcoal were well represented in the calculus matrix of the Mondeval de Sora individual. Within the starch granules, large granules (ca., 20 μm), oval to sub‐round in 2D shape, lenticular in 3D shape, with a central hilum and visible lamellae were the most abundant. In the Mondeval sample, such morphotypes of granules occurred as single grains (Figure 3,10–14) and, in one case, as in bimodal distribution, that is, with large circular granules with a clear central hilum and a higher density of deep lamellae toward the middle portion (also known as Type‐A) appearing together with small almost spherical grains with a central hilum (≤10 μm, known as Type‐B; Figure 3,9). In our reference collection, such features appear more consistently in starch granules of the genus *Aegilops* within the Triticeae tribe of the Poaceae grass family. A total of 21 starch granules assigned to morphotype 1 were found in the sample of calculus from Mondeval de Sora. Few type‐A granules also appeared damaged and having lost their original birefringence, possibly due to enzymatic damage or mechanical processing of the starch. Extensive work on 13 species of the genus *Aegilops* (*Aegilops triuncialis*, *A. comosa*, *A. crassa*, *A. ovata*, *A. biuncialis*, *A. cilidrica*, *A. speltoides*, *A. neglecta*, *A. columnaris*, *A. tawskii*, *A. peregrina*, *A. caudata*, *A. geniculata*), native of the Eastern Alpine region and the Balkan peninsula, was conducted for previous and current analyses of dental calculus (Cristiani et al., 2016; Cristiani et al., 2018; Nava et al., in press). In our reference collection, the dimensions of Type A starch granule of *Aegilops* can vary from 10 μm in some species (e.g., in *A. ovata and A. neglecta*) to more than 30 μm in other (e.g., *A. triuncialis*, *A. peregrina*, *A. geniculate*, *A. caudalis*, etc.). Based on our reference collection, and excluding any modern contamination in the analyzed sample or in the laboratory, we are confident in stating that the dimensions of the archeological starch granules (ca., 20 μm) recovered in the Mondeval de Sora dental calculus (Figure 3, 9–14) fall within the range of various species of the *Aegilops* genus. In order to provide a solid basis to our claim about the variability of *Aegilops* starch granules dimensions, we decided to add a figure with the starch granules from different *Aegilops* species from our reference collection (see Figure S3).

Together with the morphotype above, several small irregular starch granules, other plant structures were identified in the calculus (Figure 3, 15–18) as well as animal remains. In particular, numerous barbule fragments, consistent with birds of the Anatidae family based on the shape, features, and distance of their nodes, were also found (Figure 3, 19–20).

### 3.4 Dental wear pattern

**FIGURE 4:**
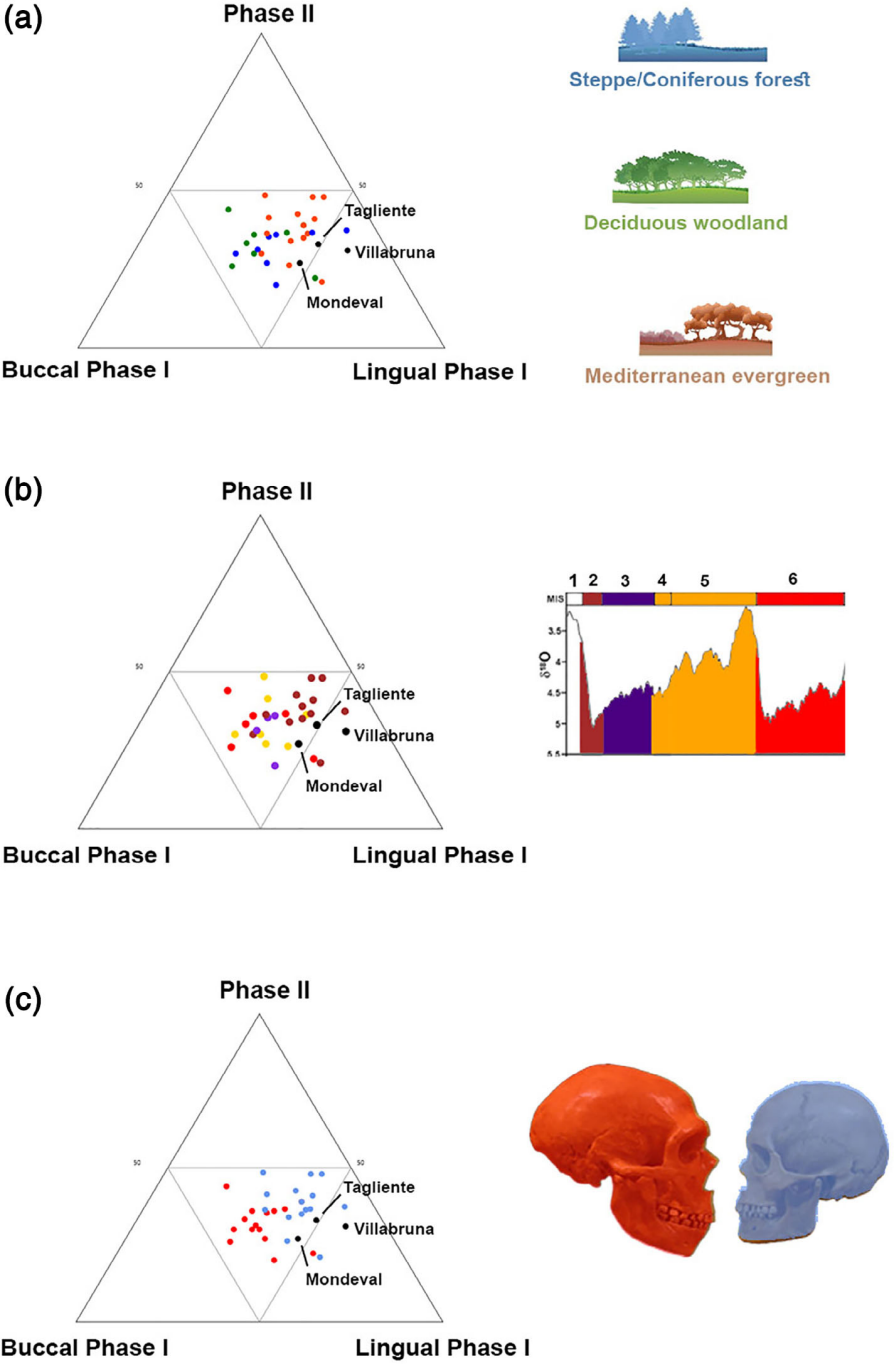
Ternary diagrams showing the proportions (in %) of relative wear surface of Buccal Phase I areas, Lingual Phase I areas, and Phase II areas (Kay & Hiiemae, 1974), which are positioned in an equilateral triangle. Each base of the triangle represents a ratio of 0% while the vertices correspond to a percentage of 100%. Our three individuals (Tagliente, Villabruna, and Mondeval) were identified in the ternary plots as black points. (a) Ecozones‐based group; (b) MIS‐based group; (c) species‐based group (Red: *Homo neanderthalensis*, blue: *Homo sapiens*)

Relative wear areas of the three individuals are characterized by high values of Lingual Phase I facets (Table S3). Mondeval (BPI: 0.27) exhibits the lowest value of Buccal Phase I wear when compared to Tagliente 2 (BPI: 0.33) and Villabruna (BPI: 0.31), as opposed to the trend observed for Phase II facets (Figure 4a).

In order to understand the segregation which emerged among our individuals, we compared the three specimens with a reference sample (Table S1) created ex novo, comprising both Neanderthal and modern human M_2_s in wear stage 2/3 (Smith, 1984). Reference individuals are grouped based on Ecogeographic zones, MIS‐groups, geography, and species.

The comparison of Phase II proportions separated into ecogeographic zones provided no significant differences, both considering the interspecific reference sample and the intraspecific modern human specimens only (Table S4). In contrast, Buccal Phase I exhibits significant differences, although after Bonferroni correction, no significant pairwise result was obtained from posthoc Mann–Whitney tests (Table S5). These results are confirmed by the lack of population structure as measured via AMOVA (*Φ*_ST_ = 0.07, *p*‐value = .054).

No significant differences were found after correction between MIS‐based groups for any of the investigated macrowear variables (Table S6). The result once again is confirmed by the lack of population structure (*Φ*_ST_ = 0.09, *p*‐value = .09).

The impact of geography on the distribution of dental macrowear in the reference sample was also investigated (Table S7). No significant differences were found through univariate analyses, while geography explains a considerable amount of variability when all variables are considered at once (*Φ*_ST_ = 0.14, *p*‐value = .033).

**FIGURE 5:**
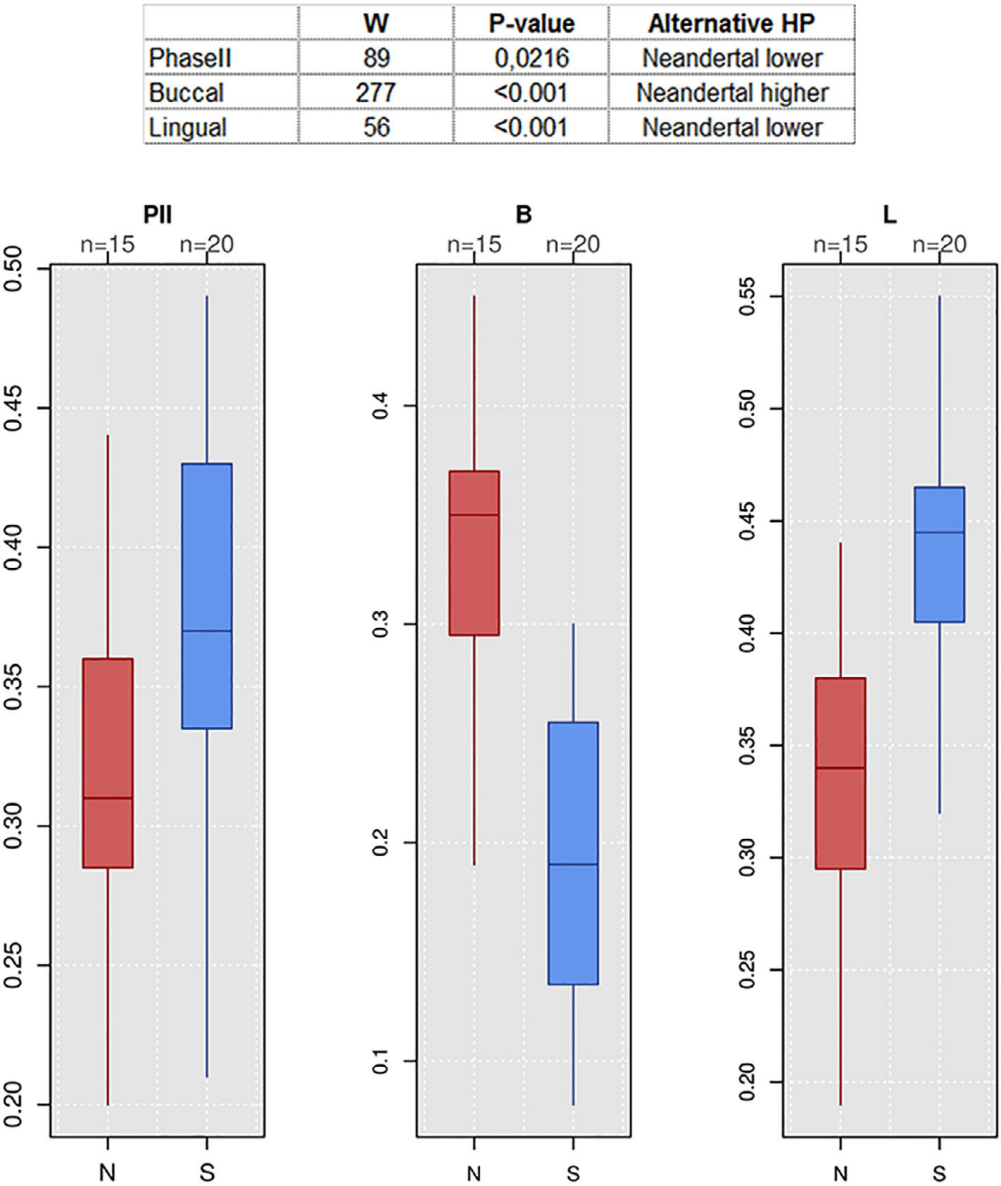
Univariate analyses result in significant differences between Neanderthal (N) and modern humans (S) for all masticatory phases (Phase II, Buccal Phase I and Lingual Phase I)

The segregation between Neanderthals and modern humans emerging from the ternary diagram (Figure 4c) is confirmed by AMOVA (*Φ*_ST_ = 0.28, *p*‐value <.001; Table S8). Univariate analyses support significant differences between Neanderthal and modern humans for all masticatory phases (Figure 5). Considering the abovementioned results, we focused on modern human specimens alone for the following analyses.

**FIGURE 6:**
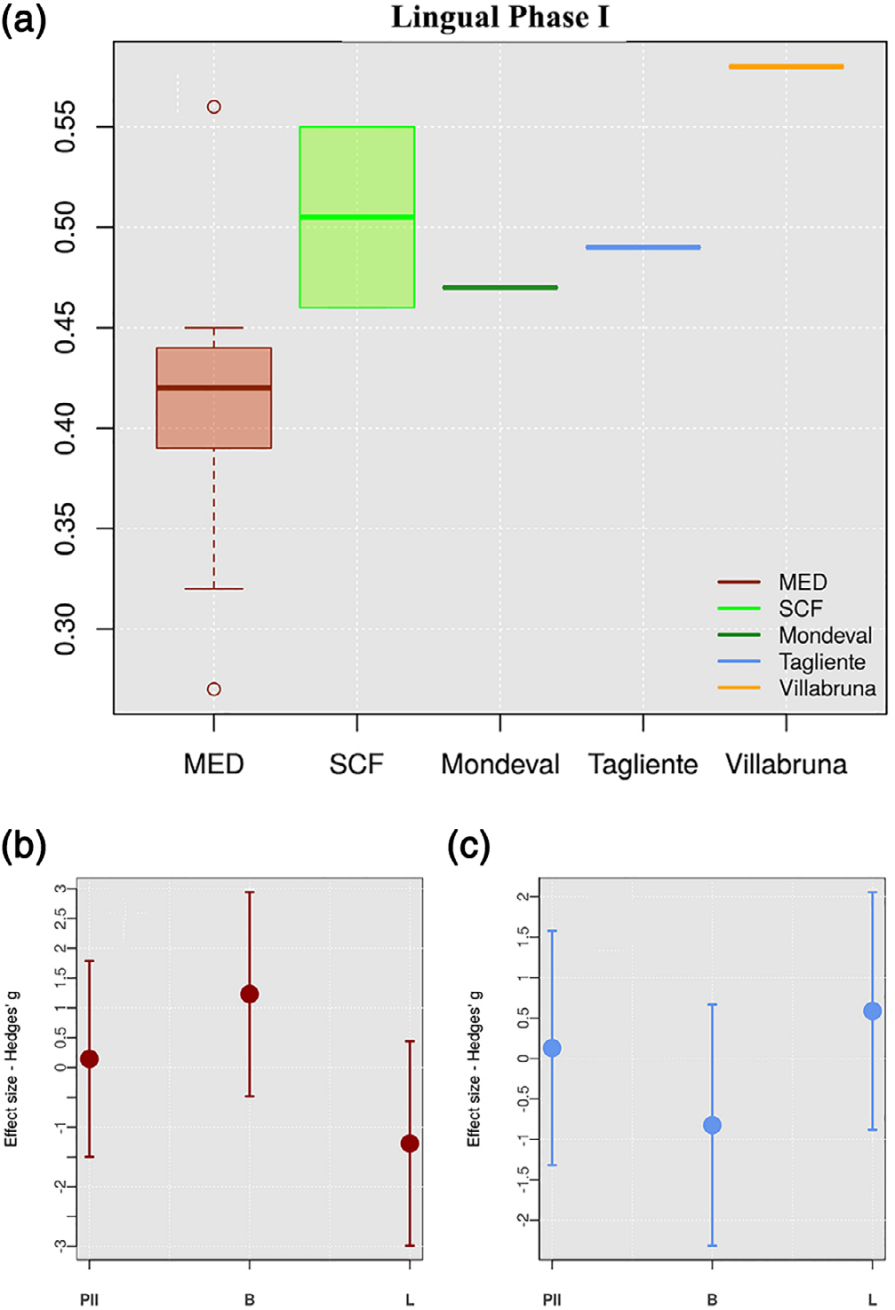
(a) Box plot of Lingual Phase I among *Homo sapiens* considering Mediterranean Evergreen (MED) and Steppe‐Coniferous Forest (SCF) ecozones. (b) Effect size between MED and SCF ecozones for each masticatory Phase In the *Homo sapiens* reference sample. (c) Effect size between MIS2 and MIS5e‐d for each masticatory Phase In the same sample

Lingual Phase I of modern humans exhibits no significant difference between individuals adapted to Mediterranean evergreen (MED) and Steppe–Coniferous forest (SCF; Mann–Whitney *W* = 2, *p*‐value = .07; Figure 6a), although effect size shows that adaptation to SCF has a more substantial impact on Buccal Phase I while adaptation to MED impacts more Lingual Phase I wear. On the other hand, a significant difference between MIS5 and MIS2 emerged for Buccal Phase I wear (*W* = 7.47, *p*‐value = .02) as suggested by statistical analysis (effect size—Figure 6 B,C).

## 4 DISCUSSION

By integrating different data from various sources, we achieved an in‐depth reconstruction of the subsistence and dietary preferences of three foragers who lived between the end of the Late Pleistocene and the Early Holocene in the Eastern Alpine region.

Overall, the isotopic analysis and the study of faunal remains indicate a significant consumption of terrestrial and, to a less extent, of aquatic animal proteins; however, the small number of faunal samples limits our interpretation.

The individual discovered at Riparo Tagliente (Tagliente 2) exhibits traces of a strong reliance on animal proteins while butchering marks on the faunal remains (Fontana et al., 2018; Fontana, Cilli, et al., 2009) point to ungulates as one of the likely sources of protein for this individual. Besides, the δ^15^N level measured in human remains is well above a trophic step shift (i.e., >3–5‰; Hedges & Reynard, 2007) compared to local herbivores and omnivores, suggesting an additional consumption of high trophic level animals (e.g., foxes) and/or freshwater resources. The composition of the faunal assemblage supports both hypotheses (see Bartolomei et al., 1982; Fontana, Cilli, et al., 2009; Gazzoni, 2011).

The δ^13^C value of −19.5‰ found at Riparo Tagliente (Tagliente 2) seems consistent with a typical terrestrial environment and freshwater fauna. As the δ^13^C ratio is generally predictive of a marine (higher δ^13^C) versus freshwater (lower δ^13^C) diet, the exploitation of freshwater fish seems the more conservative hypothesis for this individual, considering that Riparo Tagliente was located ca. 360 km away from the Adriatic Sea at the time of the Late Paleolithic occupation (Gazzoni et al., 2013). Accordingly, the consumption of freshwater resources has also been suggested for other Epigravettian human remains (e.g., Arene Candide, Liguria; Grotta del Romito, Calabria; and San Teodoro, Sicily).

In a wider perspective, the isotopic signature of Tagliente 2 is close to San Teodoro 7 (δ^15^N = 11.5 and δ^13^C = −19.1‰; Mannino, Di Salvo, et al. 2011) and in line with the high protein diet suggested for other Upper Paleolithic sites across Italy (Figure 2).

Interestingly, the other Epigravettian human from Riparo Tagliente (Tagliente 1) presents both higher δ^15^N (13‰) and δ^13^C (−18.4‰), indicating an even stronger reliance on aquatic resources (Gazzoni, 2011; Gazzoni et al., 2013; Lugli et al., 2019). Both these values may indicate that marine proteins cannot be entirely excluded from the diet of the individuals of Riparo Tagliente (Gazzoni, 2011; Gazzoni et al., 2013). Human displacements during the Late Glacial in Europe, as reflected in the archeological record, may cover comparable distances, even longer in Western and Central Europe (Roebroeks, 2001). Flint provenance also offers a unique opportunity for tracing Early and Early‐Late Epigravettian movements across the Po river valley (Po Plain). Remarkable evidence is provided by the reddish‐brownish chert contained in the Upper Cretaceous to Middle Eocene limestone‐marl formation Scaglia Rossa exploited in the Central Apennines and introduced as finished equipment in the Early Epigravettian sites of the Monti Berici and Istria during the LGM and northward to Riparo Tagliente during the early phase of the Late Epigravettian (Bertola, 2012; Bertola, Fontana, & Visentin, 2018; Peresani, 2019). However, exclusive reliance on terrestrial animals cannot be entirely ruled out for these individuals. For example, the consumption of lactating juvenile animals, cooked or putrid meat may increase the δ^15^N value above the canonical trophic level shift (see, for example, Jaouen et al., 2019).

Tagliente 1 and Tagliente 2 show a δ^13^C and a δ^15^N differences of 1.1 and 1.5, respectively. Such differences might result from the different types of samples analyzed, thus suggesting a slightly different diet between juvenile and adult individuals. In particular, stable isotopes from Tagliente 2 individual were measured on the root of the lower left first molar, the mineralization of which ends at the age of ~0 years. These isotope values thus represent a broad period of the individual's life, starting from late childhood to the adult age, due to the presence of secondary dentine, which grows during the whole life. Differences in isotopic values between Tagliente 1 and 2 might be explained in three ways: (1) Tagliente 1 and 2 belong to two individuals with slightly diverse dietary inputs or provenance; (2) Ultra‐filtrated collagen (Tagliente 2) versus nonultrafiltrated collagen (Tagliente 1) yielded different stable isotope results (see Jørkov, Heinemeier, & Lynnerup, 2007); (3) Tagliente 1 and 2 belong to the same individual, with bone turnover between mandible and rib (if any) as the main cause of the isotopic variation (see Fahy, Deter, Pitfield, Miszkiewicz, & Mahoney, 2017; Olsen et al., 2014).

The δ^15^N and δ^13^C analysis performed on the individual of Villabruna shows clear evidence of the consumption of terrestrial proteins, even though the intake of alternative proteins (e.g., aquatic resources) cannot be excluded. δ^15^N and δ^13^C values are too low to fit in the marine protein range (Richards & Hedges, 1999). The low δ^15^N value recorded in the deer bone collagen (1.6‰) from the same context could relate to climatic variations (e.g., physiological and/or dietary herbivore adaption to cold environments; differential volatilization rate in soil compounds or ^15^N depletion in soil regenerated following deglaciation; see Richards & Hedges, 2003 for a detailed discussion). Accordingly, δ^15^N values of herbivore fluctuated from 40 ka to the present with the lowest peaks observed during colder phases.

Human isotopic values of the Late Mesolithic individual from Mondeval de Sora suggest a strong reliance on terrestrial animal proteins again, with some potentially limited intake of fish and freshwater resources (see below). On average, the isotopic composition of herbivores from the site is −20.3 ± 1.2‰ (1*σ*) for carbon and 2.6 ± 1‰ (1*σ*) for nitrogen (*n* = 5; Gazzoni, 2011). The fact that the human collagen δ^15^N is well above a typical trophic step shift when compared to the herbivore mean (Δ_human‐herbivore_ = 6.5‰) indicates the likely additional intake of aquatic proteins for this individual (Gazzoni, 2011; Gazzoni et al., under review). However, considering a δ^13^C of −19.9‰ for the human collagen, such additional intake might be attributed to freshwater resources rather than marine resources. Freshwater resources tend to have a wider δ^13^C range of values, overlapping the values commonly observed in terrestrial ecosystems (Fuller, Müldner, Van Neer, Ervynck, & Richards, 2012; Richards & Hedges, 1999). Future sulfur isotope analyses may help to untangle this issue, aiming to understand whether fish consumption occurred at Mondeval the Sora.

**FIGURE 7:**
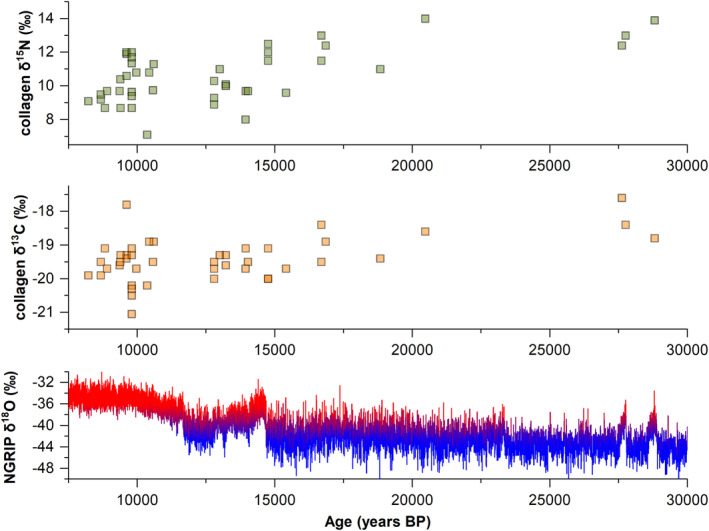
Carbon and nitrogen stable isotope values (see Table S2) are plotted over time for Upper Paleolithic and Mesolithic hunter‐gatherers from Italy. Oxygen isotope data from NGRIP (Rasmussen et al., 2014) are also reported to show the climatic variability

Overall, the isotopic data obtained for the three foragers reveal a diet primarily based on terrestrial animal proteins with contribution from freshwater and possibly marine resources. A similar pattern was already suggested for other Late Glacial and Mesolithic humans from Italy (Mannino & Richards, 2018). Overall, dietary data available for Mediterranean foragers (including Spain, Croatia and Corsica) indicate a heavy reliance on terrestrial proteins (Mannino & Richards, 2018). The use of aquatic resources is often patchy and mostly observed in groups along the Atlantic (see Richards & Hedges, 1999) or during later phases of the Mesolithic (see Cristiani et al., 2018; Mannino et al., 2015). No specific dietary shift is detected isotopically between late Upper Paleolithic and early Mesolithic. Yet, a long‐term trend (from ca., 30 to 8 ka) of both δ^15^N and δ^13^C values is evident in human collagen data (Figure 7). Such a pattern may have resulted from (a) global and/or local climatic variations, affecting plants and animal physiology, or behavior (see, for example, Bocherens, Drucker, & Madelaine, 2014; Iacumin, Nikolaev, & Ramigni, 2000 and Hedges, Stevens, & Richards, 2004); (b) a lack of isotopic data for some specific periods (e.g., only three Gravettian individuals were studied from Italian contexts); (c) a dietary shift connected to climatic adaption. Concerning the latter hypothesis, the only three Gravettian hunter‐gatherers for whom isotopic data are available display both high δ^15^N and δ^13^C values, indicating a more substantial reliance on aquatic (maybe marine) resources.

On the contrary, the lowest isotopic values are registered during the Holocene, possibly suggesting that Mesolithic individuals consumed primarily terrestrial foods, with a marginal role of aquatic foodstuff. Accordingly, our Mesolithic individual (Mondeval de Sora) shows isotope values compatible with a predominant terrestrial diet. However, the presence of a harpoon among the grave goods and the δ^15^N value slightly above one‐trophic step shift may suggest a role of freshwater resources in the diet (Hedges & Reynard, 2007). In the calculus of the Mondeval individual, feather barbules belonging to birds consistent with the *Anatidae* family were also recovered, supporting the possible consumption of such aquatic resource (as indicated by stable isotopes). This being said, the possibility that the feather fragments could have been ingested after the extra‐masticatory use of the teeth cannot be ruled out (e.g., using teeth as a tool while hafting weapons, and/or in the production of ornaments). Higher exploitation of aquatic proteins as a seasonal resource occurred during the Late Glacial phases of climatic instability (Mannino & Richards, 2018).

Dental calculus and dental macrowear analyses provide a more balanced picture of the diet of Eastern Alpine foragers, underlining a possible contribution of plant species as food. In particular, starch granules belonging to grass grains known to be of dietary importance were recovered in the analyzed dental calculi, hence providing the first, direct evidence that local foragers consumed vegetal resources during their life. Interestingly, a higher familiarity with starchy foods is documented for Mondeval de Sora forager compared to the other individuals (Figure 3). However, we cannot exclude that these results are related to the different amounts of mineralized deposit analyzed in this work.

To date, there is very little paleobotanical evidence documenting the consumption of vegetal foods is available for the investigated periods and region. Bulbous oat grass (*Arrhenatherum elatius var. bulbosum*), elder (cf., *Sambucus* sp*. L*.), and juniper fruits (cf., *Juniperus* sp*. L*.) were likely part of the Epigravettian diet at Riparo Dalmeri, in the Trentino region (Carra, Marinval, & Dalmeri, 2011). Hazelnuts (*Corylus avellana*) weren consumed during the Late Paleolithic and Mesolithic at Isola Santa (Apuanian Alps), Monte Bagioletto (Reggio Appennins), Monte Cornizzolo (western Lombard Prealps). Wild berries, such as juniper cornelian cherry, blackthorns and bearberry, are documented at Grotta di Pozzo, in Abruzzo (Castelletti, D'Errico, & Leoni, 1993; Castelletti & Leoni, 1984; Leoni, Castelletti, & Castiglioni, 2002; Mussi et al., 2000). In Sicily, wild legumes (*Lathyruys* sp., Pisum sp.), acorns (Quercus sp.), arboreal fruits (*Arbutus unedo*), and wild grapes (*Vitis silvestris*) have been found in the Mesolithic layers of Grotta dell'Uzzo (Costantini, 1989). Interestingly, direct evidence of Late Epigravettian and Mesolithic foragers' familiarity with species of grass grains of the genus *Aegilops* and the Tribe Paniceae comes from the dental calculi of individuals buried at Grotta Continenza (Nava et al., submitted). Our results further support the idea that Paleolithic and Mesolithic foragers consumed carbohydrate‐rich grass grains despite their low recovery rate in preagrarian contexts, in the absence of specific modalities of conservation (i.e., carbonization) and systematic flotation activity (see, e.g., Divišová & Šída, 2015 for an overview of the main plant remains recovered in Mesolithic Europe).

Together with dental calculus, patterns of dental macrowear from molars support a possible reliance on relatively abrasive plant foods, as signaled by high values of Lingual Phase I (Fiorenza, 2015; Lucas, 2004) wear. A previous analysis of upper molars of recent hunter‐gatherers revealed a wear pattern dominated by Buccal Phase I facets in meat‐eater human groups, and a pattern with large Lingual Phase I facets in groups of individuals who relied on mixed food resources, including a large percentage of plants (Fiorenza, Benazzi, Tausch, et al., 2011). A more recent study, however, has shown a mirrored occlusal relationship in the macrowear pattern of upper and lower molars in an Australian Aboriginal population from Yuendumu (Oxilia et al., 2018).

According to this comparative information, the macrowear patterns of the two Italian Upper Paleolithic and one Late Mesolithic individuals suggest a diet mainly based on meat with only a moderate intake of plant foods. Interestingly, at Grotta dell'Uzzo and Grotta della Molara, both in Sicily, dental microwear analysis carried out on Mesolithic individuals confirms plant intake as a component of the local diet (Borgognini Tarli & Repetto, 1985). A similar pattern is also visible in the ternary diagram (Figure 4), where Villabruna, Riparo Tagliente, and Mondeval de Sora individuals plot within the range of variability attributed to Neanderthals and modern humans from the MED group (and partly near the SCF group). Previous analyses of these groups suggested the exploitation of various food sources (Fiorenza, Benazzi, Tausch, et al., 2011), including large proportions of meat, as documented in Australian Aboriginal populations that consumed large quantities of animal proteins (O'Dea et al., 1991). When only modern humans are observed, univariate exploratory results suggest that all three individuals exhibit high values of Lingual wear consistent with SCF and out of the range of individuals adapted to MED ecozones (Figure 4a).

In particular, the Villabruna sample seems to be closer to steppe coniferous forest sample groups, as suggested by the distribution pattern of macrowear (Figure 4a), and from faunistic and palaeoenviromental data. In accordance with previous radiocarbon dating (Alciati, 1992), Villabruna falls in the fossil group that lived during MIS 2 (Figure 4b), while Neanderthals are clustered on the opposite side of the ternary plot (Figure 4c).

The ecogeographic zone that roughly defines the environment in which Tagliente 2 individual lived is attributable to steppe coniferous forest (Figure 4a), in agreement with the relevant zooarchaeological findings. Similar to the individual from Villabruna, the forager from Tagliente 2 falls closer to humans of the MIS 2 stage (Figure 4b).

The Mondeval de Sora individual presents similar Lingual macrowear values (Figure 4a). This result cannot be supported or refuted on the basis of the available zooarcheological evidence. As far as MIS‐based grouping is concerned, the Mondeval individual falls closer to humans of MIS 2 stage in the ternary diagram. However, its coordinates are closer to those of individuals adapted to warmer climates (Figure 4b). This is consistent with the direct radiocarbon dating performed on the individual (8,377–8,067 cal BP) (Fontana et al., 2016) as well as with paleoenvironmental data available for the site (Fontana, Govoni, et al., 2009).

The reference sample analyzed in this study provides additional information suggesting the possibility of discriminating between Neanderthals and modern humans by observing the distribution of macrowear in M_2_s. Considering, however, that the present reference sample is conservative and too small to generate inference at a population level, future studies should be focused on a wider sample to confirm this signal.

To sum up, through the application of a multi‐proxy approach, we successfully unraveled additional details on the dietary strategies of three hunter‐gatherers, who lived in the Eastern Alpine region between the late Upper Paleolithic and the end of the Mesolithic.

Epigravettian individuals (Tagliente and Villabruna) practiced specialized hunting and exploited wider variety of biological resources linked to the local biodiversity, and including terrestrial as well as freshwater resources. This conclusion is also supported by the dominance of species that lived in open environments and adapted to milder conditions in the associated faunal assemblages. In accordance with the still scanty paleobotanical data available for the region, plant foods were also part of the Epigravettian and Mesolithic diet, as demonstrated by starch granules identified from dental calculus and documented by Lingual wear dominance from macrowear.

The intense colonization of the central‐eastern Italian Alpine region, which occurred during the final transition and the early Holocene, was promoted by climatic oscillation, which produced forests increasing up to 2.250 m asl. More balanced exploitation of available resources (animal proteins and plant foods) was reflected by the dietary spectrum of the individual discovered at Mondeval de Sora. Human isotopic values suggest a strong reliance on terrestrial animal and freshwater resources supplemented by plant foods, the latter documented by macrowear and possibly by dental calculus analyses.

## 5 CONCLUSION

Data discussed in this article support the idea that foragers who lived in the Eastern Alpine region during the Late Paleolithic and Mesolithic were familiar with animal, freshwater as well as plant resources, hence suggesting a quite versatile human adaptation to the local environment.

The isotope data primarily emphasized the contribution of animal proteins in the prehistoric diet. However, the combination of macrowear data, dental calculus results and paleobotanical information, by stressing the potential familiarity of forager diet with plant‐foods, underlines the significance of multiple proxies for obtaining a more exhaustive picture of prehistoric foragers' diet. Besides, macrowear study generated a considerable amount of information, including the potential differences between Neanderthals and modern humans in patterns distribution on the occlusal surfaces on M_2_s.

## AUTHOR CONTRIBUTIONS

**Gregorio Oxilia:** Conceptualization; data curation; formal analysis; investigation; methodology; project administration; resources; software; supervision; validation; visualization; writing‐original draft; writing‐review and editing. **Eugenio Bortolini:** Formal analysis; investigation; methodology; software; writing‐original draft; writing‐review and editing. **Federica Badino:** Investigation; methodology; writing‐original draft; writing‐review and editing. **Federico Bernardini:** Methodology; software; visualization; writing‐original draft; writing‐review and editing. **Valentina Gazzoni:** Data curation; investigation; methodology. **Federico Lugli:** Data curation; formal analysis; investigation; methodology; writing‐original draft; writing‐review and editing. **Matteo Romandini:** Investigation; writing‐original draft; writing‐review and editing. **Anita Radini:** Data curation; investigation; methodology; visualization; writing‐original draft; writing‐review and editing. **Gabriele Terlato:** Investigation; methodology; writing‐original draft; writing‐review and editing. **Giulia Marciani:** Writing‐review and editing. **Sara Silvestrini:** Writing‐review and editing. **Jessica C. Menghi Sartorio:** Formal analysis; investigation; methodology; software; visualization; writing‐original draft; writing‐review and editing. **Ursula Thun Hohenstein:** Investigation; methodology; writing‐original draft; writing‐review and editing. **Luca Fiorenza:** Investigation; methodology; supervision; visualization; writing‐original draft; writing‐review and editing. **Ottmar Kullmer:** Investigation; methodology; supervision; writing‐original draft; writing‐review and editing. **Claudio Tuniz:** Formal analysis; investigation; methodology; visualization; writing‐original draft; writing‐review and editing. **Jacopo Moggi‐Cecchi:** Formal analysis; investigation; supervision; writing‐original draft; writing‐review and editing. **Sahra Talamo:** Methodology; software; writing‐original draft; writing‐review and editing. **Federica Fontana:** Data curation; investigation; supervision; writing‐original draft; writing‐review and editing. **Marco Peresani:** Data curation; supervision; writing‐original draft; writing‐review and editing. **Stefano Benazzi:** Funding acquisition; software; supervision; writing‐original draft; writing‐review and editing. **Emanuela Cristiani:** Conceptualization; data curation; formal analysis; funding acquisition; investigation; methodology; project administration; resources; supervision; validation; visualization; writing‐original draft; writing‐review and editing.

## CONFLICT OF INTEREST

The authors declare no competing interests.

## Supporting information


**Appendix** S1: Supporting Information

## Data Availability

The data that supports the findings of this study are available in the supplementary material of this article.

## References

[ajpa24128-bib-0001] Aimar, A. , Broglio, A. , Castelletti, L. , D'Amico, C. , Giacobini, G. , Maspero, A. , & Peresani, M. (1992). Les Abris Villabruna dans la Vallèe du Cismon. Preistoria Alpina, 28(1), 227–254.

[ajpa24128-bib-0002] Alciati, G. (1992). Mondeval de Sora: A highaltitude Mesolithic campsite in the Italian Dolomites. Preistoria Alpina, 28(1), 351–366.

[ajpa24128-bib-0003] Alciati, G. , Cattani, L. , Fontana, F. , Gerhardinger, E. , Guerreschi, A. , Milliken, S. , … Rowley‐Conwy, P. (1994). Mondeval de Sora: A high altitude Mesolithic camp‐site in the Italian Dolo‐mites. Preistoria Alpina, 28(1), 351–366.

[ajpa24128-bib-0004] Arnaud, J. , Peretto, C. , Panetta, D. , Tripodi, M. , Fontana, F. , Arzarello, M. , … Benazzi, S. (2016). A reexamination of the middle Palaeolithic human remains from Riparo Tagliente, Italy. Quaternary International, 425, 437–444.

[ajpa24128-bib-0005] Arrighi, S. , Moroni, A. , Tassoni, L. , Boschin, F. , Badino, F. , Bortolini, E. , … Benazzi, S. (2019). Bone tools, ornaments and other unusual objects during the middle to upper Palaeolithic transition in Italy. Quaternary International, (in press). 10.1016/j.quaint.2019.11.016

[ajpa24128-bib-0006] Avigliano, R. , Di Anastasio, G. , Improta, S. , Peresani, M. , & Ravazzi, C. (2000). A new late glacial ‐ early Holocene palaeobotanical and archaeological record in the eastern pre‐Alps: The Palughetto basin (Cansiglio plateau, Italy). Journal of Quaternary Science, 15(8), 789–803.

[ajpa24128-bib-0007] Baldoni, M. , Scorrano, G. , Gismondi, A. , D'Agostino, A. , Alexander, M. , Gaspari, L. , … Martínez‐Labarga, C. (2018). Who were the miners of Allumiere? A multidisciplinary approach to reconstruct the osteobiography of an Italian worker community. PLoS One, 13(10), e0205362. 30308078 10.1371/journal.pone.0205362 PMC6181348

[ajpa24128-bib-0008] Bartolomei, G. , Broglio, A. , Cattani, L. , Cremaschi, M. , Guerreschi, A. , Mantovani, E. , … Sala, B. (1982). I depositi würmiani del Riparo Tagliente. Annali dell'Università di Ferrara (N.S.), 15(III(4)), 61–105.

[ajpa24128-bib-0009] Bartolomei, G. , Broglio, A. , Guerreschi, A. , Leonardi, P. , Peretto, C. , & Sala, B. (1974). Una sepoltura epigravettiana nel deposito pleistocenico del Riparo Tagliente in Valpantena (Verona). Rivista di Scienze Preistoriche, 29(1), 101–152.

[ajpa24128-bib-0010] Bazzanella, M. , Betti, L. , Wierer, U. , (2007). *Mesolithic Wetland Exploitation at Galgenbühel/dos de la Forca*, *Italy*, *Eastern Alps. The Fish Fauna*. In Hüster Plogmann, H. (Ed.), Proceedings of the13th Fish Remains Working Group Meeting, ICAZ. Verlag Marie Leidorf Gmbh, Rahden/Westf. p. 93e100

[ajpa24128-bib-0011] Been, E. , Hovers, E. , Ekshtain, R. , Malinski‐Buller, A. , Agha, N. , Barash, A. , … Barzilai, O. (2017). The first Neanderthal remains from an open‐air middle Palaeolithic site in the Levant. Scientific Reports, 7, 2958. 28592838 10.1038/s41598-017-03025-z PMC5462778

[ajpa24128-bib-0012] Berto, C. , Luzi, E. , Guerreschi, A. , Fontana, F. , & Valletta, F. (2016). Small mammals from Mondeval de Sora (san Vito di Cadore, Belluno): Paleoenvironmental differences between early and late Holocene. Preistoria Alpina, 48, 69–72.

[ajpa24128-bib-0013] Berto, C. , Luzi, E. , Montanari Canini, G. , Guerreschi, A. , & Fontana, F. (2018). Climate and landscape in Italy during late Epigravettian. The late glacial small mammal sequence of Riparo Tagliente (Stallavena di Grezzana, Verona, Italy). Quaternary Science Reviews, 184, 132–142.

[ajpa24128-bib-0014] Bertola, S. (2012). Approccio micropaleontologico discriminante per riconoscere la provenienza alpina o appenninica delle selci della scaglia rossa. Bulletin of the Museum of Prehistoric Anthropology of Monaco, 52, 17–27.

[ajpa24128-bib-0015] Bertola S. , Broglio A. , Cassoli P. , Cilli C. , Cusinato A. , Dalmeri G. , …, Ziggiotti S. , (2007). L'Epigravettiano recente nell'area prealpina e alpina orientale. In L'Italia tra 15.000 e 10.000 anni fa. Cosmopolitismo e regionalità nel Tardoglaciale, cur. F. Martini, 39–94. Firenze: Mus. Fiorent. Preist. “Paolo Graziosi”, Stud. Archeol. Preist. 5

[ajpa24128-bib-0016] Bertola, S. , Fontana, F. , & Visentin, D. (2018). Lithic raw material circulation and settlement dynamics in the upper Palaeolithic of the venetian Prealps (NE Italy). A key‐role for palaeoclimatic and landscape changes across the LGM? In V. Borgia & E. Cristiani (Eds.), Palaeolithic Italy. Advanced studies on early human adaptation in the Apennine peninsula (pp. 219–246). Leiden: Sidestone Press.

[ajpa24128-bib-0017] Bocherens, H. , Billiou, D. , Patou‐Mathis, M. , Bonjean, D. , Otte, M. , & Mariotti, A. (1997). Paleobiological implications of the isotopic signatures (13 C, 15 N) of fossil mammal collagen in Scladina cave (Sclayn, Belgium). Quateranry Research, 48, 370–380.

[ajpa24128-bib-0018] Bocherens, H. , Drucker, D. G. , & Madelaine, S. (2014). Evidence for a 15N positive excursion in terrestrial foodwebs at the middle to upper Palaeolithic transition in South‐Western France: Implications for early modern human palaeodiet and palaeoenvironment. Journal of Human Evolution, 69, 31–43. 24630359 10.1016/j.jhevol.2013.12.015

[ajpa24128-bib-0019] Borgognini Tarli, S. , Repetto, E. (1985) Diet, dental features and oral pathology in the Mesolithic samples from Uzzo and Molara caves (Sicily). Papers in Italian Archaeology, *British archaeological reports (international series)*, Oxford. p. 245.

[ajpa24128-bib-0020] Broglio, A. (1980). Culture e ambienti della fine del Paleolitico e del Mesolitico nell'Italia Nord‐Orientale. Preistoria Alpina, 16, 7e29.

[ajpa24128-bib-0021] Broglio, A. (2001). I valichi alpini in et a paleolitica e meso‐ litica. Atti del Convegno “Uso dei valichi alpini orientali dalla preistoria ai pellegrinaggi medievali”. *Fondazione Giovanni Angelini. Centro Studi sulla Montagna*. Cason, pp. 29–5.

[ajpa24128-bib-0022] Broglio, A. & Improta, S. (1994–1995). Nuovi dati di cronologia assoluta del Paleolitico superiore e del Mesolitico del Veneto, del Trentino e del Friuli in Atti Istituto Veneto Scienze, Lettere Arti, CLIII. pp. 1–45.

[ajpa24128-bib-0023] Broglio, A. , & Lanzinger, M. (1990). Considerazioni sulla distribuzione dei siti tra la fine del Paleolitico superiore e l'inizio del Neolitico nell'Italia nord‐orientale. In: P. BIAGI (a cura di), The Neolithisation of the Alpine Region. Monografie di Natura Bresciana, 13, 53–69.

[ajpa24128-bib-0024] Broglio, A. & Villabruna, A. (1991). Vita e morte di un cacciatore di 12000 anni fa. Risultati preliminari degli scavi nei ripari Villabruna (Valle del Cismon. Val Rosna, Sovramonte, Belluno). Odeo Olimpico, pp. 1–19.

[ajpa24128-bib-0025] Bronk Ramsey, C. (2017). Methods for summarizing radiocarbon datasets. Radiocarbon, 59(2), 1809–1833.

[ajpa24128-bib-0026] Bronk Ramsey, C. , Higham, T. F. G. , Owen, D. C. , Pike, A. W. G. , & Hedges, R. E. M. (2002). Radiocarbon dates from the Oxford AMS system: Archaeometry datelist 31. Archaeometry, 44(3) Supplement I, 1–149 Printed in Great Britain.

[ajpa24128-bib-0027] Caricola, I. , Zupancich, A. , Moscone, D. , Mutri, G. , Falcucci, A. , Duches, R. , … Cristiani, E. (2018). An integrated method for understanding the function of macro‐lithic tools. Use wear, 3D and spatial analyses of an early upper Palaeolithic assemblage from north eastern Italy. PLoS One, 13(12), e0207773. 10.1371/journal.pone.0207773 30540784 PMC6291187

[ajpa24128-bib-0028] Carra, M. , Marinval, P. , & Dalmeri, G. (2011). I bulbi di avena altissima (*Arrhenatherum elatius var. bulbosum*) da Riparo Dalmeri (TN): offerta votiva o cibo quotidiano? Preistoria Alpina, 45, 147–157.

[ajpa24128-bib-0029] Castelletti, L. , D'Errico, F. & Leoni, L. , (1993). Il sito mesolitico di Monte Cornizzolo (Prealpi Lombarde Occidentali). Preisto‐ ria Alpina 19, Museo Tridentino di Scienze Naturali, Trento. pp. 213–220.

[ajpa24128-bib-0030] Castelletti, L. & Leoni, L. , (1984). Carboni di Bagioletto Alto. In M. Cremaschi, et al. *Il sito mesolitico di Monte Bagioletto (Appennino Reggiano) nel quadro delle variazioni ambien‐ tali oloceniche dell'Appennino Tosco‐Emiliano* (Vol. 9, pp. 38–44). Emilia Pre‐romana.

[ajpa24128-bib-0031] Clark, R. (2000). The Mesolithic hunters of the Trentino. A case study in hunter‐gatherer settlement and subsistence from northern Italy. BAR International Series, 832, 210.

[ajpa24128-bib-0032] Collina, C. , Marciani, G. , Martini, I. , Donadio, C. , Repola, L. , Bortolini, E. , … Benazzi, S. (2020). Refining the Uluzzian through a new lithic assemblage from Roccia san Sebastiano (Mondragone, southern Italy). Quaternary International, (In press). 10.1016/j.quaint.2020.03.056

[ajpa24128-bib-0033] Colombo, L. , Martinelli, E. , Motella, S. , Castelletti, L. , Fontana, F. , Guerreschi, A. , & Michetti, A. (2016). A contribution to landscape reconstruction in the basin of Mondeval de Sora (Belluno Dolomites, N‐E Italy): Preliminary analysis of an anthracological sample from the Mesolithic layers of site VF1, sectors I and III. Preistoria Alpina, 48, 245–253.

[ajpa24128-bib-0034] Corrain, C. (1966). Un frammento di mandibola umana, rinvenuto al RiparoTagliente in Valpantena (Verona). Atti dell'Istituto Veneto di Scienze, Lettere Ed Arti. 1965–66.

[ajpa24128-bib-0035] Corrain, C. (1977). I resti scheletrici umani della sepoltura epigravettiana del Riparo Tagliente in Valpantena (Verona). Bollettino del Museo Civico di Storia Naturale di Verona, 4, 35–79.

[ajpa24128-bib-0036] Costantini, L. (1989). Plant exploitation at Grotta dell'Uzzo, Sicily: New evidence for the transition from Mesolithic to Neolithic subsistence in southern Europe. In D. Harris & G. Hillman (Eds.), Foraging and farming. the evolution of plant exploitation. London and New York: Routledge Library Editions.

[ajpa24128-bib-0037] Craig, O. E. , Biazzo, M. , Colonese, A. C. , Di Giuseppe, Z. , Martinez‐Labarga, C. , Lo Vetro, D. , … Rickards, O. (2010). Stable isotope analysis of late upper Palaeolithic human and faunal remains from Grotta del Romito (Cosenza), Italy. Journal of Archaeological Science, 37, 2504–2512.

[ajpa24128-bib-0038] Cristiani, E. , Radini, A. , Borić, D. , Robson, H. K. , Caricola, I. , Carra, M. , … Vujević, D. (2018). Dental calculus and isotopes provide direct evidence of fish and plant consumption in Mesolithic Mediterranean. Scientific Reports, 8(1), 8147. 29802341 10.1038/s41598-018-26045-9 PMC5970156

[ajpa24128-bib-0170] Cristiani, E., Radini, A., Edinborough, M., & Borić, D. (2016). Dental calculus reveals mesolithic foragers in the balkans consumed domesticated plant foods. *Proceedings of the National Academy of Sciences of the United States of America*, 113(37), 10298–10303. 10.1073/pnas.1603477113 PMC5027412 27573829

[ajpa24128-bib-0039] Crompton, A. W. , & Hiiemae, K. M. (1970). Functional occlusion and mandibular movements during occlusion in the American opossum, *Didelphis marsupialis* . Zoological Journal of the Linnean Society, 49, 21–47.

[ajpa24128-bib-0040] Di Maida, G. , Mannino, M. A. , Krause‐Kyora, B. , Jensen, T. Z. T. , & Talamo, S. (2019). Radiocarbon dating and isotope analysis on the purported Aurignacian skeletal remains from Fontana Nuova (Ragusa, Italy). PLoS One, 14(3), e0213173. 30893326 10.1371/journal.pone.0213173 PMC6426221

[ajpa24128-bib-0041] Divišová, M. , & Šída, P. (2015). Plant use in the Mesolithic period. Archaeobotanical data from The Czech Republic in a European context–A review. Interdisciplinaria Archaeologica: Natural Sciences in Archaeology, 6(1), 95–106.

[ajpa24128-bib-0042] Dove, C. J. (1998). Feather evidence helps clarify locality of anthropological artifacts in the Museum of Mankind. Pacific Studies, 21(3), 73–85.

[ajpa24128-bib-0188] Dove, C. J., & Agreda, A. (2007). Differences in plumulaceous feather characters of dabbling and diving ducks. *The Condor* , 109(1), 192–199.

[ajpa24128-bib-0043] Dove, C. J. , & Koch, S. (2011). Microscopy of feathers: A practical guide for forensic feather identification. Microscope, 59(2), 51–71.

[ajpa24128-bib-0193] Drescher‐Schneider, R. (2009). La storia forestale delle alpi sud‐orientali e del margine pedemontano durante gli ultimi 25 mila anni. Le Foreste Dei Cacciatori Paleolitici. Ambiente E Popolamento Umano in Cansiglio Tra Tardoglaciale E Postglaciale, pp. 27e64. Supplemento Al Bollettino Della Societa Naturalisti Silvia Zenari. Pordenone.

[ajpa24128-bib-0044] Drucker, D. , & Bocherens, H. (2004). Carbon and nitrogen stable isotopes as tracers of change in diet breadth during middle and upper Palaeolithic in Europe. International Journal of Osteoarchaeology, 14(3–4), 162–177.

[ajpa24128-bib-0045] El Zaatari, S. , & Hublin, J. J. (2014). Humans: Evidence from microwear texture analysis. American Journal of Physical Anthropology, 153(4), 570–581. 10.1002/ajpa.22457 24449141

[ajpa24128-bib-0046] Excoffier, L. , Smouse, P. E. , & Quattro, J. M. (1992). Analysis of molecular variance inferred from metric distances among DNA haplotypes: Application to human mitochondrial DNA restriction data. Genetics, 131(2), 479–491. 1644282 10.1093/genetics/131.2.479 PMC1205020

[ajpa24128-bib-0047] Fahy, G. E. , Deter, C. , Pitfield, R. , Miszkiewicz, J. J. , & Mahoney, P. (2017). Bone deep: Variation in stable isotope ratios and histomorphometric measurements of bone remodelling within adult humans. Journal of Archaeological Science, 87, 10–16.

[ajpa24128-bib-0048] Figus, C. , Traversari, M. , Scalise, L. M. , Oxilia, G. , Vazzana, A. , Buti, L. , … Benazzi, S. (2017). The study of commingled non‐adult human remains: Insights from the 16th–18th centuries community of Roccapelago (Italy). Journal of Archaeological Science: Reports, 14, 382–391.

[ajpa24128-bib-0049] Fiore, I. , & Tagliacozzo, A. (2005). Lo sfruttamento delle risorse animali nei siti di altura e di fondovalle nel Tardiglaciale dell'Italia nord‐orientale. In G. Malerba & P. Visentini (Eds.), Quaderni Museo Archeologico Friuli Occidentale, (pp. 97–109). Pordenone: Comune di Pordenone.

[ajpa24128-bib-0050] Fiore, I. , & Tagliacozzo, A. (2006). Lo sfruttamento dello stambecco nel Tardiglaciale di Riparo Dalmeri (TN): il livello 26c. In U. Tecchiati & B. Sala (Eds.), Studi di Archeozoologia in onore di Alfredo Riedel, (pp. 59–76). Bolzano: Ufficio Beni Culturali.

[ajpa24128-bib-0051] Fiorenza, L. (2015). Reconstructing diet and behaviour of Neanderthals from Central Italy through dental macrowear analysis. Journal of Archaeological Science, 93, 1–15. 10.4436/JASS.93002 25324463

[ajpa24128-bib-0052] Fiorenza, L. , Benazzi, S. , Henry, A. G. , Salazar‐García, D. C. , Blasco, R. , Picin, A. , … Kullmer, O. (2015). To meat or not to meat? New perspectives on Neanderthal ecology. American Journal of Physical Anthropology, 156(S59), 43–71. 25407444 10.1002/ajpa.22659

[ajpa24128-bib-0053] Fiorenza, L. , Benazzi, S. , & Kullmer, O. (2009). Morphology, wear and 3‐D digital surface models: Materials and techniques to create high resolution replicas of teeth. Journal of Anthropological Science, 87, 211–218. 19663176

[ajpa24128-bib-0054] Fiorenza, L. , Benazzi, S. , & Kullmer, O. (2011). Para‐masticatory wear facets and their functional significance in hunter‐gatherer maxillary molars. Journal of Archaeological Science, 38(9), 2182–2189.

[ajpa24128-bib-0055] Fiorenza, L. , Benazzi, S. , Oxilia, G. , & Kullmer, O. (2018). Functional relationship between dental macrowear and diet in late Pleistocene and recent modern human populations. International Journal of Osteoarchaeology, 28(2), 1–9.

[ajpa24128-bib-0056] Fiorenza, L. , Benazzi, S. , Tausch, J. , Kullmer, O. , Bromage, T. G. , & Schrenk, F. (2011). Molar macrowear reveals Neanderthal eco‐geographical dietary variation. PLoS One, 6(3), e14769. 21445243 10.1371/journal.pone.0014769 PMC3060801

[ajpa24128-bib-0057] Fiorenza, L. , & Kullmer, O. (2013). Dental wear and cultural behaviour in middle Palaeolithic humans from the near east. American Journal of Physical Anthropology, 152(1), 107–117. 23904240 10.1002/ajpa.22335

[ajpa24128-bib-0058] Fiorenza, L. , & Kullmer, O. (2015). Dental wear patterns in early modern humans from Skhul and Qafzeh: A response to Sarig and Tillier. Homo, 66(5), 414–419. 26048367 10.1016/j.jchb.2015.04.002

[ajpa24128-bib-0059] Floris, R. , Melis, R. T. , Mussi, M. , Palombo, M. R. , & Iacumin, P. (2012). La presenza umana nella Sardegna centro occidentale durante l'Olocene antico: il sito di S'Omu e S'Orku, Arbus, VS. La presenza umana nella Sardegna centro occidentale durante l'Olocene antico: il sito di S'Omu e S'Orku, Arbus, VS, pp. 999–1004.

[ajpa24128-bib-0060] Fontana, F. (2006). La sepoltura di Mondeval de Sora (Belluno). Differenziazione sociale e modalità insediative degli ultimi popoli cacciatori e raccoglitori dell'Italia nord‐orientale. In F. Martini (Ed.), *La cultura del morire nelle società preistoriche e protostoriche italiane. Studio interdisciplinare dei dati e loro trattamento informatico. Dal Paleolitico all'età del Rame*. Istituto Italiano di Preistoria e Protostoria, Origines, Progetti 3, Firenze, pp. 269–292.

[ajpa24128-bib-0061] Fontana, F. , Bertola, S. , Cremona, M. G. , Cavulli, F. , Falceri, L. , Gajardo, A. , … Guerreschi, A. (2018). Re‐colonising the southern alpine fringe: Diachronic data on the use of sheltered space in the late Epigravettian site of Riparo Tagliente. In V. Borgia & E. Cristiani (Eds.), Palaeolithic Italy advanced studies on early human adaptation in the Apennine peninsula (pp. 287–310). Leiden: Sidestone Press.

[ajpa24128-bib-0062] Fontana, F. , Cilli, C. , Cremona, M. G. , Giacobini, G. , Gurioli, F. , Liagre, J. , … Guerreschi, A. (2009). Recent data on the Late Epigravettian occupation at Riparo Tagliente, Monti Lessini (Grezzana, Verona): a multidisciplinary perspective. Preistoria Alpina, 44, 51–59 (ISSN 0393‐0157).

[ajpa24128-bib-0063] Fontana, F. , Govoni, L. , Guerreschi, A. , Padoanello, S. , Siviero, A. , Thun Hohenstein, U. , & Ziggiotti, S. (2009). L'occupazione sauveterriana di Mondeval de Sora 1, settore I (San Vito di Cadore, Belluno) in bilico tra accampamento residenziale e campo da caccia. Preistoria Alpina, 44, 207–226.

[ajpa24128-bib-0064] Fontana, F. & Guerreschi, A. (2003). *Highland Occupation in the Southern Alps in Mesolithic on the Move*. In L. Larsson (ed.). Proceedings of the 6th International Conference on the Meso‐lithic in Europe. 4th–8th September 2000 Stockholm. Oxbow Books, pp. 96–102.

[ajpa24128-bib-0065] Fontana, F. , Guerreschi A. , Bertola S. , Briois F. , & Ziggiotti S. (2016). *The Castelnovian Burial of Mondeval de Sora (San Vito di Cadore*, *Belluno*, *Italy): Evidence for Changes in the Social Organization of Late Mesolithic Hunter‐Gatherers in North‐Eastern Italy*. In Grünberg J., Gramsch B., Larsson L., Orschiedt J, & Meller H. (Eds.). *Mesolithic burials – Rites*, *symbols and social organisation of early postglacial communities*. Proceedings of the International Conference, Halle, 18–21, September 2013. pp. 741–756.

[ajpa24128-bib-0066] Fontana, F. , Guerreschi, A. , Pasi, E. , & Petrucci, G. (2009). Premiers résultats sur l'étude des niveaux sauveterriens du site 1, secteur III de Mondeval de Sora (Dolomites, Belluno, Italie). Rivista di Scienze Preistoriche, LIX, 79–92.

[ajpa24128-bib-0067] Fontana, F. , & Visentin, D. (2016). Early Mesolithic highland and lowland occupation between the venetian Alps and the Emilian Apennines (northern Italy). Quaternary International, 423, 266–278.

[ajpa24128-bib-0068] Fontana, F. , & Vullo, N. (2000). Organisation et fonction d'un camp de base saisonnier au coeur des Dolomites: le gisement mésolithique de Mondeval de Sora (Belluno, Italie). *Actes du Colloque International Epipaléolithique Mésolithique. Les derniers chasseurs d'Europe occidentale*, 23–25 Ottobre 1998. pp. 197–208.

[ajpa24128-bib-0069] Francalacci, P. , & Tarli, S.B. . (1988). *Multielementary Analysis of Trace Elements and Preliminary Results on Stable Isotopes in Two Italian Prehistoric Sites. Methodological Aspects*. In Grupe, G. & Herrmann, B (Eds.). *Trace Elements in Environmental History*. Proceedings in Life Sciences. Berlin, Heidelberg: Springer.

[ajpa24128-bib-0070] Fuller, B. T. , Müldner, G. , Van Neer, W. , Ervynck, A. , & Richards, M. P. (2012). Carbon and nitrogen stable isotope ratio analysis of freshwater, brackish and marine fish from Belgian archaeological sites (1 st and 2 nd millennium AD). Journal of Analytical Atomic Spectrometry, 27(5), 807–820.

[ajpa24128-bib-0071] Gaudzinski‐Windheuser, S. , & Kindler, L. (2012). The evolution of hominin food resource exploitation in Pleistocene Europe: Recent studies in zooarchaeology. Quaternary International, 252, 1–2.

[ajpa24128-bib-0072] Gazzoni, V. (2011). Contributo alla ricostruzione delle identità regionali e della differenziazione sociale presso i gruppi di cacciatori‐raccoglitori paleo‐mesolitici. Studio della ritualità funeraria in Italia e Francia e analisi degli isotopi stabili sul campione umano del versante alpino sud‐orientale. Tesi di Dottorato, Università degli Studi di Ferrara.

[ajpa24128-bib-0073] Gazzoni, V. , Goude, G. , Dalmeri, G. , Guerreschi, A. , Mottes, E. , Nicolis, F. , … Fontana, F. (under review). Investigating diet of Mesolithic groups in the southern Alps: An attempt through stable carbon and nitrogen isotope analyses. Bulletin de la Société d'Anthropologie de Paris.

[ajpa24128-bib-0074] Gazzoni, V. , Goude, G. , Herrscher, E. , Guerreschi, A. , Antonioli, F. , & Fontana, F. (2013). Late upper Palaeolithic human diet: First stable isotope evidence from Riparo Tagliente (Verona, Italy). Bulletins et mémoires de la Société d'anthropologie de Paris, 25, 103–117.

[ajpa24128-bib-0075] Gerhardinger, E. , & Guerreschi, A. (2006)*. La découverte d*'*une sépulture mésolithique à Mondeval de Sora (Belluno*, *Italie*). In Hominidae. Proceedings of the 2nd International Congress of Human Palaeontology (Milano 1989) pp. 511–513.

[ajpa24128-bib-0076] Germonpré, M. , Sablin, M. , Khlopachev, G. A. , & Grigorieva, G. V. (2008). Possible evidence of mammoth hunting during the Epigravettian at Yudinovo, Russian plain. Journal of Anthropological Archaeology, 27, 475–492.

[ajpa24128-bib-0077] Gismondi, A. , Baldoni, M. , Gnes, M. , Scorrano, G. , D'Agostino, A. , Di Marco, G. , … Martínez‐Labarga, C. (2020). A multidisciplinary approach for investigating dietary and medicinal habits of the medieval population of Santa Severa (7^th^‐15^th^ centuries, Rome, Italy). PLoS One, 15, e0227433. 10.1371/journal.pone.0227433 31990948 PMC6986732

[ajpa24128-bib-0078] Grippo, J. O. , Simring, M. , & Schreiner, S. (2004). Attrition, abrasion, corrosion and abreaction revisited: A new perspective on tooth surface lesions. Journal of the American Dental Association, 135(8), 1109–1118. 15387049 10.14219/jada.archive.2004.0369

[ajpa24128-bib-0079] Harwood, H. P. (2011). Identification and description of feathers in Te Papa's of the Museum of New Zealand Te Papa Tongarewa (Vol. 22, pp. 125–147).

[ajpa24128-bib-0080] Hedges, R. E. , & Reynard, L. M. (2007). Nitrogen isotopes and the trophic level of humans in archaeology. Journal of Archaeological Science, 34(8), 1240–1251.

[ajpa24128-bib-0081] Hedges, R. E. , Stevens, R. E. , & Richards, M. P. (2004). Bone as a stable isotope archive for local climatic information. Quaternary Science Reviews, 23(7–8), 959–965.

[ajpa24128-bib-0082] Henry, A. G. , Brooks, A. S. & Piperno, D. R. (2011). Microfossils in calculus demonstrate consumption of plants and cooked foods in neanderthal diets (Shanidar III, Iraq; Spy I and II, Belgium. *Proceedings of the National Academy* of *Sciences, USA*, *108*, 486–491. 10.1073/pnas.1016868108 PMC3021051 21187393

[ajpa24128-bib-0083] Henry, A. G. , Hudson, H. F. , & Piperno, D. R. (2009). Changes in starch grain morphologies from cooking. Journal of Archaeological Science, 36(3), 915–922.

[ajpa24128-bib-0084] Hiiemäe, K. M. , & Crompton, A. W. (1971). A cinefluorographic study of feeding in the American opossum, *Didelphis marsupialis*. In A. A. Dahlberg (Ed.), Dental morphology and evolution (pp. 299–334). Chicago: University Chicago Press.

[ajpa24128-bib-0085] Hiiemae, K. M. , & Kay, R. F. (1972). Trends in the evolution of primate mastication. Nature, 240, 486487. 10.1038/240486a0 4565949

[ajpa24128-bib-0086] Iacumin, P. , Nikolaev, V. , & Ramigni, M. (2000). C and N stable isotope measurements on Eurasian fossil mammals, 40 000 to 10 000 years BP: Herbivore physiologies and palaeoenvironmental reconstruction. Palaeogeography, Palaeoclimatology, Palaeoecology, 163(1–2), 33–47.

[ajpa24128-bib-0087] Ivy‐Ochs, S. , Kerschner, H. , Reuther, A. , Preusser, F. , Heine, K. , Maisch, M. , … Schluchter, C. (2008). Chronology of the last glacial cycle in the European Alps. Journal of Quaternary Science, 23(6–7), 559–573.

[ajpa24128-bib-0088] Jaouen, K. , Richards, M. P. , Le Cabec, A. , Welker, F. , Rendu, W. , Hublin, J. J. , … Talamo, S. (2019). Exceptionally high δ^15^N values in collagen single amino acids confirm Neandertals as high‐trophic level carnivores. Proceedings of the National Academy of Sciences, 166(11), 4928–4933. 10.1073/pnas.1814087116 PMC6421459 30782806

[ajpa24128-bib-0089] Jørkov, M. L. S. , Heinemeier, J. , & Lynnerup, N. (2007). Evaluating bone collagen extraction methods for stable isotope analysis in dietary studies. Journal of Archaeological Science, 34(11), 1824–1829.

[ajpa24128-bib-0090] Kay, R. F. (1977). The evolution of molar occlusion in the Cercopithecidae and early catarrhines. American Journal of Physical Anthropology, 46(2), 327–352. 848569 10.1002/ajpa.1330460213

[ajpa24128-bib-0091] Kay, R. F. , & Hiiemae, K. M. (1974). Jaw movement and tooth use in recent and fossil primates. American Journal of Physical Anthropology, 40(2), 227–256. 4815136 10.1002/ajpa.1330400210

[ajpa24128-bib-0092] Kimoto, K. , Fushima, K. , Tamaki, K. , Toyoda, M. , Sato, S. , & Uchimura, N. (2000). Asymmetry of masticatory muscle activity during the closing phase of mastication. Cranio, 18(4), 257–263. 11202845 10.1080/08869634.2000.11746139

[ajpa24128-bib-0093] Kullmer, O. , Benazzi, S. , Fiorenza, L. , Schulz, D. , Bacso, S. , & Winzen, O. (2009). Technical note: Occlusal fingerprint analysis: Quantification of tooth wear pattern. American Journal of Physical Anthropology, 139(4), 600–605. 19425091 10.1002/ajpa.21086

[ajpa24128-bib-0094] Lambeck, K. , Rouby, H. , Purcell, A. , Sun, Y. , & Sambridge, M. (2014). Sea level and global ice volumes from the last glacial maximum to the Holocene. Proceedings of the National Academy of Sciences of the United States of America, 111, 15296–15303. 25313072 10.1073/pnas.1411762111 PMC4217469

[ajpa24128-bib-0095] Leoni L. , Castelletti L. & Castiglioni E. , 2002. I carboni epigravet‐tiani e mesolitici e la dinamica della copertura forestale a Iso‐la Santa. Rivista di Scienze Preistoriche LII, Firenze, pp. 183–195.

[ajpa24128-bib-0096] Longin, R. (1971). New method of collagen extraction for radiocarbon dating. Nature, 230(5291), 241–242. 4926713 10.1038/230241a0

[ajpa24128-bib-0097] Lucas, P. W. (2004). Dental functional morphology. How teeth work. Cambridge: Cambridge University Press.

[ajpa24128-bib-0098] Lugli, F. , Cipriani, A. , Capecchi, G. , Ricci, S. , Boschin, F. , Boscato, P. , … Ronchitelli, A. (2019). Strontium and stable isotope evidence of human mobility strategies across the last glacial maximum in southern Italy. Nature Ecology and Evolution, 3(6), 905–911. 31086279 10.1038/s41559-019-0900-8

[ajpa24128-bib-0099] Lussi, A. (2006). Dental erosion: From diagnosis to therapy (Vol. 20). Basel, Switzerland: Karger Medical and Scientific Publishers.

[ajpa24128-bib-0100] Maier, W. , & Schneck, G. (1981). Konstruktionsmorphologische Untersuchungen am Gebiß der hominoiden Primaten. Zeitschrift für Morphologie Und Anthropologie, 72, 127–169. 7314796

[ajpa24128-bib-0101] Mannino, M. , & Richards, M. P. (2018). The role of aquatic resources in ‘Italian’ hunter‐gatherer subsistence and diets. In V. Borgia & E. Cristiani (Eds.), Palaeolithic Italy (pp. 397–426). Oxford: Sidestone Press Academics.

[ajpa24128-bib-0102] Mannino, M. A. , Catalano, G. , Talamo, S. , Mannino, G. , Di Salvo, R. , Schimmenti, V. , … Richards, M. P. (2012). Origin and diet of the prehistoric hunter‐gatherers on the Mediterranean Island of Favignana (Ègadi islands, Sicily). PLoS One, 7(11), e49802. 23209602 10.1371/journal.pone.0049802 PMC3509116

[ajpa24128-bib-0103] Mannino, M. A., Di Salvo, R., Schimmenti, V., Di Patti, C., Incarbona, A., Sineo, L., & Richards, M. P. (2011). Upper Palaeolithic hunter‐gatherer subsistence in Mediterranean coastal environments: An isotopic study of the diets of the oldest directly‐dated humans from Sicily. *Journal of Archaeological Science* , 38, 3094–3100

[ajpa24128-bib-0104] Mannino, M. A. , Talamo, S. , Tagliacozzo, A. , Fiore, I. , Nehlich, O. , Piperno, M. , & Richards, M. P. (2015). Climate‐driven environmental changes around 8,200 years ago favoured increases in cetacean strandings and Mediterranean hunter‐gatherers exploited them. Scientific Reports, 5, 16288. 26573384 10.1038/srep16288 PMC4648091

[ajpa24128-bib-0105] Mannino, M. A. , Thomas, K. D. , Leng, M. J. , Di Salvo, R. , & Richards, M. P. (2011). Stuck to the shore? Investigating prehistoric hunter‐gatherer subsistence, mobility and territoriality in a Mediterranean coastal landscape through isotope analyses on marine mollusc shell carbonates and human bone collagen. Quaternary International, 244(1), 88–104.

[ajpa24128-bib-0106] Marciani, G. , Ronchitelli, A. , Arrighi, S. , Badino, F. , Bortolini, E. , Boscato, P. , … Benazzi, S. (2019). Lithic techno‐complexes in Italy from 50 to 39 thousand years BP: An overview of lithic technological changes across the middle‐upper Palaeolithic boundary. Quaternary International, (in press). 10.1016/j.quaint.2019.11.005

[ajpa24128-bib-0107] Margherita, C. , Oxilia, G. , Barbi, V. , Panetta, D. , Hublin, J. J. , Lordkipanidze, D. , … Benazzi, S. (2017). Morphological description and morphometric analyses of the upper Palaeolithic human remains from Dzudzuana and Satsurblia caves, western Georgia. Journal of Human Evolution, 113, 83–90. 29054170 10.1016/j.jhevol.2017.07.011

[ajpa24128-bib-0108] Margherita, C. , Talamo, S. , Wiltschke‐Schrotta, K. , Senck, S. , Oxilia, G. , Sorrentino, R. , … Benazzi, S. (2016). A reassessment of the presumed Torrener Bärenhöhle's Palaeolithic human tooth. Journal of Human Evolution, 93, 120–125. 26976744 10.1016/j.jhevol.2016.01.007

[ajpa24128-bib-0109] Metcalf, J. L. , Ursell, L. K. , & Knight, R. (2014). Ancient human oral plaque preserves a wealth of biological data. Nature Genetics, 46(4), 321–323. 24675519 10.1038/ng.2930

[ajpa24128-bib-0110] Molnar, S. , & Molnar, I. M. (1990). Dental arch shape and tooth wear variability. American Journal of Physical Anthropology, 82, 385–395. 2375386 10.1002/ajpa.1330820314

[ajpa24128-bib-0111] Monegato, G. , Scardia, G. , Hajdas, I. , Rizzini, F. , & Piccin, A. (2017). The alpine LGM in the boreal ice‐sheets game. Scientific Reports, 7, 2078. 28522806 10.1038/s41598-017-02148-7 PMC5437061

[ajpa24128-bib-0112] Montoya, C. , Duches, R. , Fontana, F. , Peresani, M. , & Visentin, D. (2018). Peuplement tardiglaciaire et holocène ancien des Préalpes de la Vénétie (Italie Nord Orientale): éléments de confrontation in L'Aquitaine à la fin des temps glaciaires. Les sociétés de la transition du Paléolithique final au début du Mésolithique dans l'espace Nord aquitain in Actes de la table ronde organisée en hommage à Guy Célérier, Les Eyzies‐de‐Tayac, 24–26 juin 2015, PALEO, numéro spécial. Averbouh, A., Bonnet‐Jacquement, P. & Cleyet‐Merle, J. (Eds.), es Eyzies de Tayac. Musée national de Préhistoire, pp. 193–202.

[ajpa24128-bib-0113] Mussi, M. , Coubray, S. , Giraudi, C. , Mazzella, G. , Toniutti, P. , Wilkens, B. & Zampetti, D . 2000. L'exploitation des territoires de montagne dans les Abruzzes (Italie centrale) entre le Tardigla‐ ciaire et l'Holocène ancien. In: Actes de la Table ronde. Epi‐ paléolithique et Mésolithique. Lausanne, 21–23 novembre, 1997, Chahiers d'archéologie romande, Vol. 81, pp. 277–284.

[ajpa24128-bib-0114] Mussi, M. , & Peresani, M. (2011). Human settlement of Italy during the younger Dryas. Quaternary International, 242(2), 360–370.

[ajpa24128-bib-0115] Naudinot, N. A. , Tomasso, A. , Tozzi, C. , & Peresani, M. (2014). Changes in mobility patterns as a factor of 14C date density variation in the late Epigravettian of northern Italy and southeastern France. Journal of Archaeological Science, 52, 578–590.

[ajpa24128-bib-0116] Nava, A. , Fiorin, E. , Zupancich, A. , Carra, M. L. , Ottoni, C. , di Carlo, G. , … Cristiani, E. (submitted). Multipronged dental analyses reveal dietary differences in last foragers and first farmers at Grotta Continenza, Central Italy (15,500–7000 BP). PLoS One. 10.1038/s41598-021-82401-2 PMC7895915 33608594

[ajpa24128-bib-0117] O'Dea, K. , Jewell, P. A. , Whiten, A. , Altmann, S. A. , Strickland, S. S. , & Oftedal, O. T. (1991). Traditional diet and food preferences of Australian aboriginal hunter‐gatherers. Philosophical Transactions of the Royal Society B Biological Sciences, 334, 233–241. 10.1098/rstb.1991.0112 1685581

[ajpa24128-bib-0118] Oeggl, K. , & Wahlmüller, N. (1994). Vegetation e climate history of a high alpine Mesolithic camp site in the eastern Alps. Preistoria Alpina, 28(1), 71–82.

[ajpa24128-bib-0119] Olsen, K. C. , White, C. D. , Longstaffe, F. J. , von Heyking, K. , McGlynn, G. , Grupe, G. , & Rühli, F. J. (2014). Intraskeletal isotopic compositions (δ13C, δ15N) of bone collagen: Nonpathological and pathological variation. American Journal of Physical Anthropology, 153(4), 598–604. 24374993 10.1002/ajpa.22459

[ajpa24128-bib-0120] Oxilia, G. , Bortolini, E. , Martini, S. , Papini, A. , Boggioni, M. , Buti, L. , … Benazzi, S. (2018). The physiological linkage between molar inclination and dental macrowear pattern. American Journal of Physical Anthropology, 166(4), 941–951. 29633246 10.1002/ajpa.23476 PMC6120545

[ajpa24128-bib-0121] Oxilia, G. , Fiorillo, F. , Boschin, F. , Boaretto, E. , Apicella, S. A. , Matteucci, C. , … Benazzi, S. (2017). The dawn of dentistry in the late upper Palaeolithic: An early case of pathological intervention at Riparo Fredian. American Journal of Physical Anthropology, 163(3), 446–461. 28345756 10.1002/ajpa.23216

[ajpa24128-bib-0122] Oxilia, G. , Peresani, M. , Romandini, M. , Matteucci, C. , Debono Spitieri, C. , Henry, A. G. , … Benazzi, S. (2015). Earliest evidence of dental caries manipulation in the late upper Palaeolithic. Scientific Reports, 5, 12150. 26179739 10.1038/srep12150 PMC4504065

[ajpa24128-bib-0123] Pellegrini, C. , Maselli, V. , Cattaneom, A. , Piva, A. , Ceregato, A. , & Trincardi, F. (2015). Anatomy of a compound delta from the post‐glacial transgressive record in the Adriatic Sea. Marine Geology, 362, 43–59.

[ajpa24128-bib-0124] Peresani, M. , 2019. *Settling a No‐Mans Land. An Up‐Dated Rewiev on the Peopling of Northern Italy at the Last Glacial Maximum*. In Schmidt, I., Cascalheira, J., Bicho, N., & Weniger, G.C., (Eds.), 3rd International Conference on the Solutrean, 2017, Faro, Cambridge Scholars Publishing, pp. 26–43.

[ajpa24128-bib-0125] Peresani, M. , Bertola, S. , De Stefani, M. , & Di Anastasio, G. (1999–2000). Bus de La Lum and the Epigravettian occupation of the venetian pre‐Alps during the younger Dryas. Rivista di Scienze Preistoriche, 50, 103–132.

[ajpa24128-bib-0126] Pettitt, P. , Richards, M. , Maggi, R. , & Formicola, V. (2003). The Gravettian burial known as the prince (“Il Principe”): New evidence for his age and diet. Antiquity, 77(295), 15–19.

[ajpa24128-bib-0127] Phoca‐Cosmetatou, N. (2005a). Landscape use in Northeast Italy during the upper Palaeolithic. Preistoria Alpina, 41, 23–49.

[ajpa24128-bib-0128] Phoca‐Cosmetatou, N. (2005b). Bone weathering and food procurement strategies: Assessing the reliability of our behavioural inferences. In T. P. O'Connor (Ed.), Biosphere to lithosphere: New studies in vertebrate Taphonomy (pp. 135–145). Oxford: Oxbow Books.

[ajpa24128-bib-0129] Phoca‐Cosmetatou, N. (2009). Specialisation & diversification: A tale of two subsistence strategies from late glacial Italy. Before Farming, 3, 1–29.

[ajpa24128-bib-0130] Pini, R. (2002). A high‐resolution late glacial ‐ Holocene pollen diagram from Pian di Gembro (Central Alps, northern Italy). Vegetation History and Archaeobotany, 11(4), 251–262.

[ajpa24128-bib-0131] Radini, A. , Buckley, S. , Nikita, E. , Copeland, L. , & Hardy, K. (2017). Beyond food: The multiple pathways for inclusion of materials into ancient dental calculus. American Journal of Physical Anthropology, 162(S63), 71–83. 28105717 10.1002/ajpa.23147

[ajpa24128-bib-0132] Radini, A. , Nikita, E. , & Shillito, L. M. (2016). Human dental calculus anda Medieval urban environment. In B. Jervis , L. Broderick , & I. G. Solo‐gestoa (Eds.), Objects, environment, and everyday life in medieval Europe (pp. 297–313). Turnhout: BREPOLS, Human Dental Calculus and a Medieval Urban Environment.

[ajpa24128-bib-0154] Ramsey, C. B. (2009). Bayesian analysis of radiocarbon dates. *Radiocarbon* , 51(1), 337–360.

[ajpa24128-bib-0133] Rasmussen, S. O. , Bigler, M. , Blockley, S. P. , Blunier, T. , Buchardt, S. L. , Clausen, H. B. , … Winstrup, M. (2014). A stratigraphic framework for abrupt climatic changes during the last glacial period based on three synchronized Greenland ice‐core records: Refining and extending the INTIMATE event stratigraphy. Quaternary Science Reviews, 106, 14–28.

[ajpa24128-bib-0134] Ravazzi, C. , Pini, R. , Badino, F. , De Amicis, M. , Londeix, L. , & Reimer, P. J. (2014). The latest LGM culmination of the Garda glacier (Italian Alps) and the onset of glacial termination. Age of glacial collapse and vegetation chronosequence. Quaternary Science Reviews, 105, 26–47.

[ajpa24128-bib-0135] Reimer, P. J. , Bard, E. , Bayliss, A. , Beck, J. W. , Blackwell, P. G. , Bronk Ramsey, C. , … van der Plicht, J. (2013). IntCal13 and Marine13 radiocarbon age calibration curves 0–50,000years cal BP. Radiocarbon, 55(4).1869–1887.

[ajpa24128-bib-0136] Richards, M. P. , & Hedges, R. E. (1999). Stable isotope evidence for similarities in the types of marine foods used by late Mesolithic humans at sites along the Atlantic coast of Europe. Journal of Archaeological Science, 26(6), 717–722.

[ajpa24128-bib-0137] Richards, M. P. , & Hedges, R. E. M. (2003). Variations in bone collagen δ13C and δ15N values of fauna from Northwest Europe over the last 40 000 years. Palaeogeography, Palaeoclimatology, Palaeoecology, 193(2), 261–267.

[ajpa24128-bib-0138] Riga, A. , Oxilia, G. , Panetta, D. , Savadori, P. , Benazzi, S. , Wadley, L. , & Moggi‐Cecchi, J. (2018). Human deciduous teeth from the middle Stone age layers of Sibudu cave (South Africa). Journal of Anthropological Sciences, 96, 75–87. 30153107 10.4436/JASS.96005

[ajpa24128-bib-0139] Rocci Ris, A. (2006). *I macromammiferi di Riparo Tagliente. Archeozoologia e tafonomia dei livelli epigravettiani*. (PhD Thesis). Dottorato di ricerca in Scienze Antropologiche, Ciclo XVIII, Dipartimento di Anatomia, Farmacologia e medicina Legale, Università degli Studi di Torino, 535 p.

[ajpa24128-bib-0140] Roebroeks, W. (2001). Hominid behaviour and the earliest occupation of Europe: An exploration. Journal of Human Evolution, 41(5), 437–461. 11681861 10.1006/jhev.2001.0499

[ajpa24128-bib-0141] Romandini, M. , Peresani, M. , Gurioli, F. , & Sala, B. (2012). *Marmota marmota*, the most predated species at Grotta del Clusantin. Insights from an unusual case‐study in the Italian Alps. Quaternary International, 252, 184–194.

[ajpa24128-bib-0142] Rossato, S. (2013). Late quaternary glaciations and connections to the piedmont plain in the prealpine environment: The middle and lower Astico Valley (NE Italy). Quaternary International, 288, 8–24.

[ajpa24128-bib-0143] Sala, B. (1990). Loess fauna in deposits of shelters and caves in the Veneto region and examples in other region of Italy. In I. Cremaschi (Ed.), The loess in northern and central Italy: a loess basin between the Alps and the Mediterranean region, Quaderni di Geodinamica Alpina e Quaternaria, *1*, (pp. 139–149). Milano: Editrice Gutenberg.

[ajpa24128-bib-0144] Sameera, S. D. , Singh, D. P. , & Nitya, D. (2017). Bruxism: Its multiple causes and its effects on dental implants: A review. International Journal of Oral and Dental Health, 2, 57–63.

[ajpa24128-bib-0145] Seguinot, J. , Ivy‐Ochs, S. , Jouvet, G. , Huss, M. , Funk, M. , & Preusser, F. (2018). Modelling last glacial cycle ice dynamics in the Alps. The Cryosphere, 12(10), 3265–3285. 10.5194/tc-12-3265-2018

[ajpa24128-bib-0146] Semenov, S. A. (1964). Prehistoric technology; an experimental study of the oldest tools and artefacts from traces of manufacture and wear. (eds Cory, Adams & Mackay). The Antiquaries Journal, 45, 115–116.

[ajpa24128-bib-0147] Smith, B. H. (1984). Patterns of molar wear in hunter‐gatherers and agriculturists. American Journal of Physical Anthropology, 63(1), 39–56. 6422767 10.1002/ajpa.1330630107

[ajpa24128-bib-0148] Sorrentino, R. , Bortolini, E. , Lugli, F. , Mancuso, G. , Buti, L. , Oxilia, G. , … Benazzi, S. (2018). Unravelling biocultural population structure in 4^th^/3^rd^ century BC Monterenzio Vecchio (Bologna, Italy) through a comparative analysis of strontium isotopes, non‐metric dental evidence, and funerary practices. PLoS One, 13(3), e0193796. 10.1371/journal.pone.0193796 29590155 PMC5874009

[ajpa24128-bib-0149] Stone, H. H. (1948). Oral and dental diseases. Edinburgh: E. and S. Livingstone.

[ajpa24128-bib-0150] Stout, D. (2011). Stone toolmaking and the evolution of human culture and cognition. Philosophical Transactions of the Royal Society B: Biological Sciences, 366(1567), 1050–1059. 10.1098/rstb.2010.0369 PMC3049103 21357227

[ajpa24128-bib-0151] Terlato, G. , Bocherens, H. , Romandini, M. , Nannini, N. , Hobson, K. A. , & Peresani, M. (2018). Chronological and isotopic data support a revision for the timing of cave bear extinction in Mediterranean Europe. Historical Biology: An International Journal of Paleobiology, 31(4), 474–484.

[ajpa24128-bib-0152] Torchiano, M. (2018). Effsize: efficient effect size computation. doi: 10.5281/zenodo.1480624. Availabl from 10.5281/zenodo.1480624. R package version 0.7.4. Available from https://CRAN.R-project.org/package=effsize.

[ajpa24128-bib-0153] Maselli, V. , Trincardi, F. , Asioli, A. , Ceregato, A. , Rizzetto, F. , & Taviani, M. (2014). Delta growth and river valleys: The influence of climate and sea level changes on the south Adriatic shelf (Mediterranean Sea). Quaternary Science Reviews, 99, 146–163.

[ajpa24128-bib-0164] Thun Hohenstein, U., Turrini, M. C, Guerreschi, A., & Fontana, F. (2016). Red deer vs. ibex hunting at a seasonal base camp in the Dolomites: Mondeval de Sora, site 1, sector I. Quaternary International, 423, 92–101. 10.1016/j.quaint.2016.02.054.

[ajpa24128-bib-0155] Tuniz, C. , Bernardini, F. , Cicuttin, A. , Crespo, M. L. , Dreossi, D. , Gianoncelli, A. , … Zanolli, C. (2013). The ICTP‐Elettra X‐ray laboratory for cultural heritage and archaeology. Nuclear Instruments and Methods in Physics Research Section A: Accelerators, Spectrometers, Detectors and Associated Equipment, 711, 106–110.

[ajpa24128-bib-0156] Valletta, F. , Fontana, F. , Bertola, S. , & Guerreschi, A. (2016). The Mesolithic lithic assemblage of site VF1‐sector III of Mondeval de Sora (Belluno, Italy). Preistoria Alpina, 48, 73–81.

[ajpa24128-bib-0157] Vazzana, A. , Scalise, L. M. , Traversari, M. , Figus, C. , Apicella, S. A. , Buti, L. , … Benazzi, S. (2018). A multianalytic investigation of weapon‐related injuries in a late antiquity necropolis, Mutina, Italy. Journal of Archaeological Science: Reports, 17, 550–559.

[ajpa24128-bib-0158] Vercellotti, G. , Alciati, G. , Richards, M. , & Formicola, V. (2008). The late upper Palaeolithic skeleton Villabruna 1 (Italy): A source of data on biology and behavior of a 14.000 year‐old hunter. Journal of Archaeological Science, 86, 143–163. 19934473

[ajpa24128-bib-0159] Vescovi, E. , Ravazzi, C. , Arpenti, E. , Finsinger, W. , Pini, R. , Valsecchi, V. , … Tinner, W. (2007). Interactions between climate and vegetation on the southern side of the Alps and adjacent areas during the late‐glacial period as recorded by lake and mire sediment archives. Quaternary Science Reviews, 26(2007), 1650–1669.

[ajpa24128-bib-0160] Visentin, D. , Carrer, F. , Fontana, F. , Cavulli, F. , Frare, P. C. , Mondini, C. , & Pedrotti, A. (2016). Prehistoric landscapes of the Dolomites: Survey data from the highland territory of Cadore (Belluno Dolomites, northern Italy). Quaternary International, 402, 5–14.

[ajpa24128-bib-0161] Warinner, C. , Rodrigues, J. F. M. , Vyas, R. , Trachsel, C. , Shved, N. , Grossmann, J. , … Cappellini, E. (2014). Pathogens and host immunity in the ancient human oral cavity. Nature Genetics, 46(4), 336–344. 24562188 10.1038/ng.2906 PMC3969750

[ajpa24128-bib-0162] Weyrich, L. S. , Duchene, S. , Soubrier, J. , Arriola, L. , Llamas, B. , Breen, J. , … Cooper, A. (2017). Neanderthal behaviour, diet, and disease inferred from ancient DNA in dental calculus. Nature, 544(7650), 357–361. 28273061 10.1038/nature21674

[ajpa24128-bib-0172] Wick, L. (1994). Vegetation development and human impact at the forest limit: Palaeoecological studies in the Splügen pass area (north Italy). *Monografie di Natura Bresciana* , 20, 123–132.

[ajpa24128-bib-0163] Wierer, U. , & Boscato, P. (2006). Lo sfruttamento delle risorse animali nel sito mesolitico di Galgenbühel/Dos de la Forca, Salorno (Bz): la macrofauna. In U. Tecchiati & B. Sala (Eds.), Studi di Archeozoologia in onore di Alfredo Riedel, (pp. 85–98). Bolzano: Ufficio Beni Culturali.

[ajpa24128-bib-0194] Wißing, C. , Rougier, H. , Baumann, C. , Comeyne, A. , Crevecoeur, I. , Drucker, D. G. , … Matthies, T. (2019). Stable isotopes reveal patterns of diet and mobility in the last Neandertals and first modern humans in Europe. Scientific Reports, 9(1), 1–12. 30872714 10.1038/s41598-019-41033-3 PMC6418202

[ajpa24128-bib-0165] Yang, X. , & Perry, L. (2013). Identification of ancient starch grains from the tribe Triticeae in the North China plain. Journal of Archaeological Science, 40, 3170–3177.

