# Supplementary material for: Exploring late Paleolithic and Mesolithic diet in the Eastern Alpine region of Italy through multiple proxies
Source: Am J Phys Anthropol. 2020 Sep 11;174(2):232–53. doi: 10.1002/ajpa.24128 (PMC7918647; doi:10.1002/ajpa.24128)
Supplement: Supplementary file 1 — Appendix S1: Supporting Information [file AJPA-174-232-s001.docx]

**SUPPORTING INFORMATION**

**Exploring Late Palaeolithic and Mesolithic diet in the Eastern Alpine Region of Italy through multiple-proxies.**

Gregorio Oxilia, Eugenio Bortolini, Federica Badino, Federico Bernardini, Valentina Gazzoni, Federico Lugli, Matteo Romandini, Anita Radini, Gabriele Terlato, Giulia Marciani, Sara Silvestrini, Jessica C. Menghi Sartorio, Ursula Thun Hohenstein, Luca Fiorenza, Ottmar Kullmer, Claudio Tuniz, Jacopo Moggi Cecchi, Sahra Talamo, Federica Fontana, Marco Peresani, Stefano Benazzi, Emanuela Cristiani.

**Methods**

*Archeozoology*

Zooarchaeological analysis of the bone assemblages has been carried out on Riparo Villabruna (yielded by layers of 16 – 17), Mondeval de Sora (Layers 7 and 7II) and Riparo Tagliente (Layer 13 Layers 409-410-416-417-418-420) faunal remains. All the remains have been counted, separating the burned and calcined bones from the unburned, and grouped by size (0-1cm, 1-2cm, 2-3cm, 3-4cm, 4-5cm, >5cm). They were then examined for taxonomical attribution at a family level, in instances when species or genus level could not be determined. Taxonomic and skeletal identification was based on two reference collections in the Prehistoric and Anthropological Sciences Section at the University of Ferrara and (for Riparo Tagliente) at the Department of Anatomy, Pharmacology and Forensic Medicine of the University of Torino. In order to determine the nature of the surface bone alterations, and to distinguish human from animal traces, trampling abrasion, and modern mechanical modifications produced by excavation tools, reference was made to the well-established taphonomic literature (Kromer, Lindauer, Synal, & Wacker, 2013; Wick, 1994; Binford, 1981; Brain, 1981; Potts & Shipman, 1981; Shipman, 1981, 1984; Blumenschine & Selvaggio, 1988; Capaldo & Blumenschine, 1994; Blumenschine, 1995; Fisher, 1995). The degree of combustion was evaluated employing the methodology developed by Stiner, Kuhn, Weiner, & Bar-Yosef, (1995). Sex and age at death were determined to complete the dataset that would be used in reconstructing the strategies of exploitation of different species (Mariezkurrena, 1983, Vigal & Machordom 1985; D’Errico & Vanhaeren, 2002; Fiore & Tagliacozzo, 2006). Measurements were taken following Von Den Driesch (1976). In order to evaluate species abundance, the following methods were used: number of identified specimens (NISP) (Grayson, 1984), and the estimate of the minimum number of individuals (MNI) (Bökönyi, 1970). Estimation of deer MNI is based on the eruption and dental wear.

*Stable isotopes of Mondeval de Sora*

First, samples were mechanically cleaned using a dental power drill and washed with deionized water in a ultrasonic bath. Cleaned samples were crushed to obtain ~500 mg of powder. Then, bone powder was demineralized in a 1 M HCl solution at room temperature. After 20 min, the residue was soaked using 0.125 M NaOH for 20 h at room temperature and then solubilized in a 0.01 M HCl solution for 17 h at 100° C. Finally, the digested collagen solution was filtered and freeze-dried for 48 h. Elemental composition and isotope ratios were determined at the Iso-Analytical lab (Sandbach, UK) by elemental analyzer isotope ratio mass spectrometry (EA-IRMS; Europa Scientific 20-20 IRMS). IAEA reference materials (RMs) were employed: bovine liver (IA-R042), ammonium sulphate (IA-R045), beet sugar (IA-R005) and sugar cane (IA-R006). All samples and RMs were expressed as delta (δ) notation (‰), in relation to V-PBD for C and AIR for N. Typical reproducibility of both δ13CV-PDB and δ15NAIR was better than 0.1‰ (1σ). (Gazzoni et al. 2013).

The extracted collagen meets the standard quality parameters suggested by Van Klinken (1999). In particular, the collagen yield is higher than the 1 % threshold for low-collagen bones; moreover, all the C:N ratios (3.0-3.3) but two fall within the standard 2.9-3.6 range for well-preserved collagen (Van Klinken, 1999).

*Dental Calculus analysis*

At the laboratory, ancient samples were processed in a dedicated clean space. In order to verify the presence and nature of potential modern contamination present in the laboratory, experimental extraction activities related to our modern starch reference collection has never be conducted in proximity of the laboratory space dedicated to the calculus extraction and analysis. Also, the areas of extraction, slide mounting and the microscope table were regularly monitored by analyzing dust collected in “traps” before and during the analysis. Routine checks have never identified the presence of micro-particles comparable with the ones recovered in archeological calculi. In particular, no starch granule has been found in our control sample, which mostly included human fibers from the laboratory coats, building material dust and some palm pollens. However, none of the above debris was ever identified in any slides with archaeological samples. Control samples of soil adhering to the calculus was also available for dubious finds.

The laboratory where dental calculus analysis has been carried out is located in the Dentistry Department, an environment with a highly hygienic condition where cleaning is carried out daily. No food is allowed in the laboratory in order to prevent any type of modern contamination. The gloves used during all steps of the calculus demineralization and analysis were starch-free. Any possible contamination from Maize grains, sometime used as powder in gloves, are excluded as (1) no maize grains are not present in the reference collection of the laboratory where the calculus have been processed and analyzed; (2) no maize or Triticeae-type starch granules were ever found in the sample controls carried out in the laboratory; and (3) Ii our laboratory we have never analyzes either human or stone material from South America.


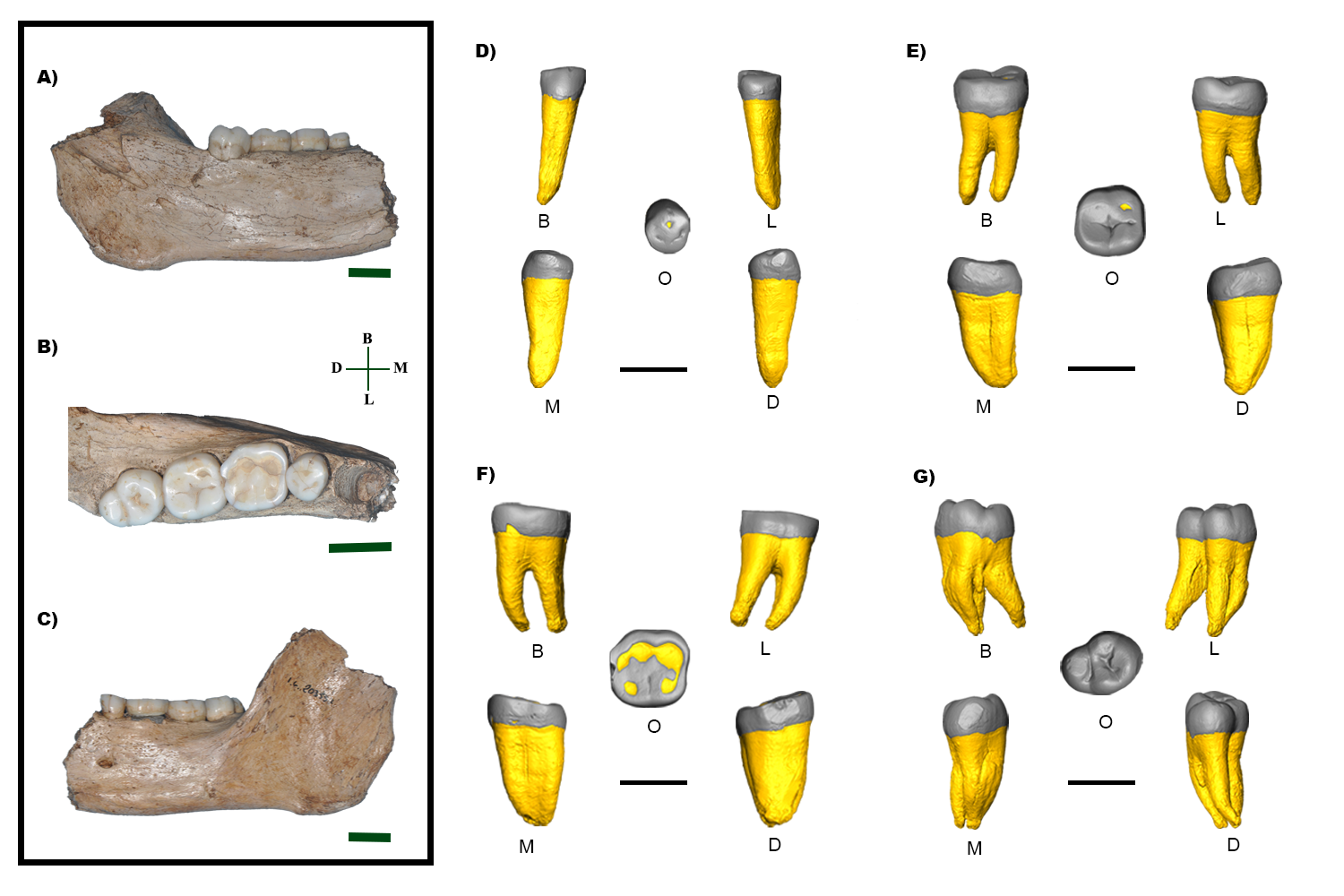
**Figure S1.** On the left the mandible discovered at the Riparo Tagliente. On the right, teeth virtually extracted.


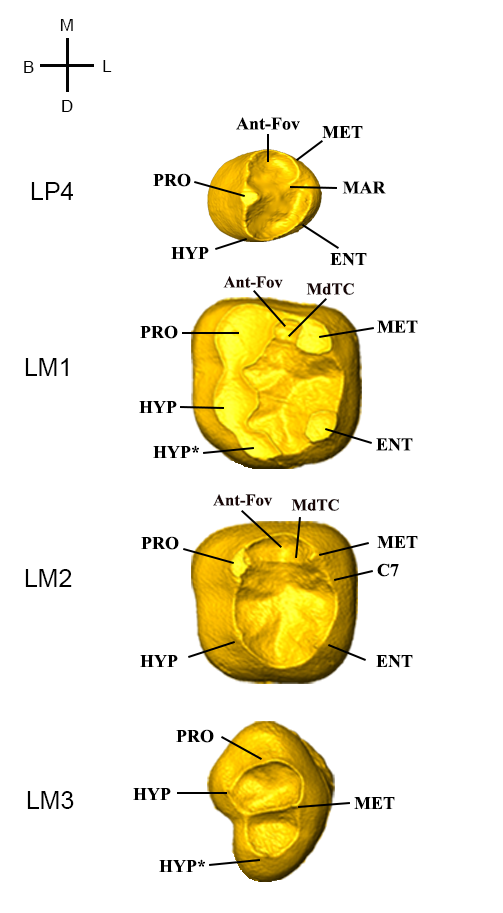


**Figure S2.** Enamel-dentine junction of teeth virtually analyzed. Ant-Fov: Anterior Fovea; MET: Metaconid; MAR: Mesial Accessory Ridge; ENT: Entoconid; HYP: Hypoconid; PRO: Protoconid; MdTC: Middle Trigonid Crest; HYP*: Hypoconulid

*
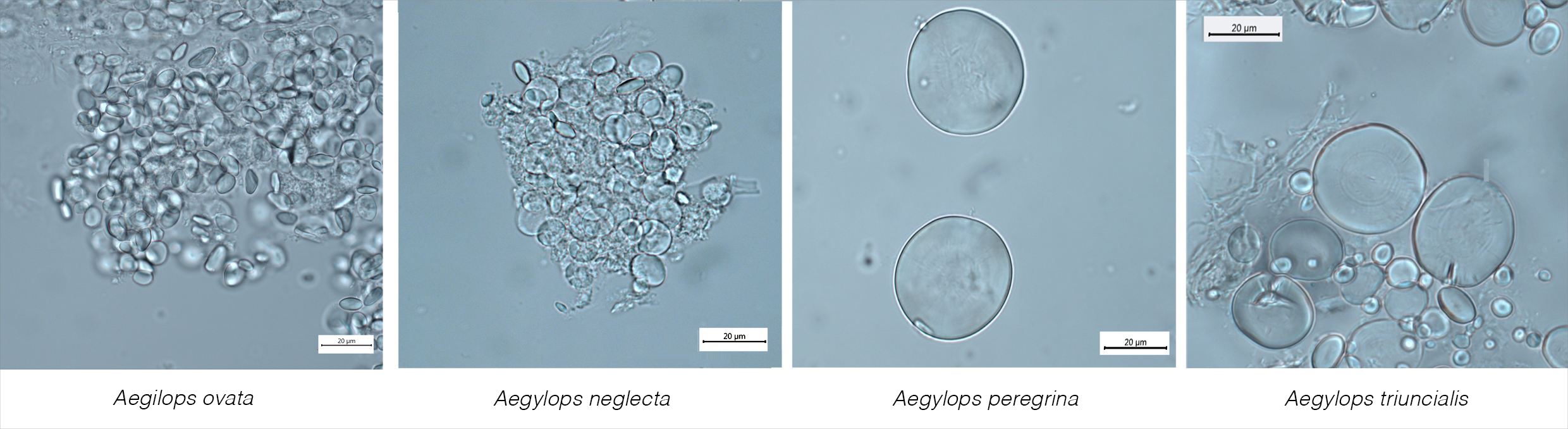
*

**Figure S3** starch granules from different species of the genus *Aegylops* from our reference collection showing the variability of starch granules dimensions within the different species.

**Table S1.** List of hunter‐gatherers and Epipalaeolithic Natufians used.

|  |  |  |  |  |  |
| --- | --- | --- | --- | --- | --- |
| **Individual** | **Specie** | **dating** | **MIS** | **Ecozone** | **Geography** |
| Krapina54 | N | 130±10 | 6_5e | DEW | Croatia |
| Krapina1 | N | 130±10 | 6_5e | DEW | Croatia |
| Krapina10 | N | 130±10 | 6_5e | DEW | Croatia |
| Krapina53 | N | 130±10 | 6_5e | DEW | Croatia |
| Krapina57 | N | 130±10 | 6_5e | DEW | Croatia |
| Krapina58 | N | 130±10 | 6_5e | DEW | Croatia |
| Krapina58a | N | 130±10 | 6_5e | DEW | Croatia |
| Krapina59 | N | 130±10 | 6_5e | DEW | Croatia |
| Krapina86 | N | 130±10 | 6_5e | DEW | Croatia |
| Krapina6 | N | 115±15 | 6_5e | DEW | Croatia |
| Subalyuk | N | 80-110 | 5d_a | SCF | Hungary |
| Tabun1 | N | 122±16 | 5e | MED | Israel |
| Arcy B9IV66 | N | 29-40 | 3 | SCF | France |
| LaQuina9 | N | 40-70 | 4 | SCF | France |
| Petit1 | N | 40-60 | 3 | SCF | France |
| SaintCesaire1 | N | 40-41 | 3 | SCF | France |
| Spy1 | N | 36 | 3 | SCF | Belgium |
| Vindija206 | N | 42 | 3 | SCF | Croatia |
| Skhul6 | MH | 100-130 | 5e_5c | MED | Israel |
| Qafzeh H27 | MH | 115±15 | 5e_d | MED | Israel |
| Qafzeh7 | MH | 115±15 | 5e_d | MED | Israel |
| Qafzeh9 | MH | 115±15 | 5e_d | MED | Israel |
| Zafarraya5_B290 | MH | 27-33 | 2 | MED | Spain |
| Oase1 | MH | 35 | 2 | SCF | Romania |
| BarmaGrande4 | MH | 24.8 | 2 | SCF | Italy |
| EM_H32 | MH | 11.5-15 | 2 | MED | Israel |
| EM_H87_l | MH | 11.5-15 | 2 | MED | Israel |
| EMH69_ | MH | 11.5-15 | 2 | MED | Israel |
| FA_H1_ | MH | 11.5-15 | 2 | MED | Israel |
| FA_H26_ | MH | 11.5-15 | 2 | MED | Israel |
| FA_H43_ | MH | 11.5-15 | 2 | MED | Israel |
| FAH8_ | MH | 11.5-15 | 2 | MED | Israel |
| HAY_H11 | MH | 11.5-15 | 2 | MED | Israel |

| **Table S2.** Collagen δ13C and δ15N of Mesolithic and Upper Palaeolithic human remains from Italy. | | | | | |
| --- | --- | --- | --- | --- | --- |
| **Site** | **Region (Italy)** | **Period** | **δ13C (‰)** | **δ15N (‰)** | **Reference** |
| Arene Candide | Liguria | Upper Palaeolithic (Gravettian) | -17,6 | 12,4 | Pettitt et al., 2003 |
| Grotta Paglicci | Apulia | Upper Palaeolithic (Gravettian) | -18,8 | 13,9 | Lugli et al., 2019 |
| Grotta Paglicci | Apulia | Upper Palaeolithic (Gravettian) | -18,4 | 13 | Lugli et al., 2019 |
| Grotta Paglicci | Apulia | Upper Palaeolithic (Epigravettian) | -19,4 | 11 | Lugli et al., 2019 |
| Grotta Paglicci | Apulia | Upper Palaeolithic (Epigravettian) | -18,6 | 14 | Lugli et al., 2019 |
| Riparo Tagliente | Veneto | Upper Palaeolithic (Epigravettian) | -18,4 | 13 | Gazzoni et al., 2013 |
| Riparo Tagliente | Veneto | Upper Palaeolithic (Epigravettian) | -19,5 | 11,5 | this study |
| Grotta del Romito | Calabria | Upper Palaeolithic (Epigravettian) | -20 | 10,3 | Craig et al., 2010 |
| Grotta del Romito | Calabria | Upper Palaeolithic (Epigravettian) | -19,3 | 10,1 | Craig et al., 2010 |
| Grotta del Romito | Calabria | Upper Palaeolithic (Epigravettian) | -19,6 | 10 | Craig et al., 2010 |
| Grotta del Romito | Calabria | Upper Palaeolithic (Epigravettian) | -19,7 | 9,3 | Craig et al., 2010 |
| Grotta del Romito | Calabria | Upper Palaeolithic (Epigravettian) | -19,5 | 8,9 | Craig et al., 2010 |
| Grotta del Romito | Calabria | Upper Palaeolithic (Epigravettian) | -19,1 | 9,7 | Craig et al., 2010 |
| Grotta del Romito | Calabria | Upper Palaeolithic (Epigravettian) | -19,5 | 9,7 | Craig et al., 2010 |
| Grotta del Romito | Calabria | Upper Palaeolithic (Epigravettian) | -18,9 | 12,4 | Craig et al., 2010 |
| Riparo Villabruna | Veneto | Upper Palaeolithic (Epigravettian) | -19,7 | 8 | Vercellotti et al., 2008 |
| Grotta Addaura | Sicily | Upper Palaeolithic (Epigravettian) | -19,7 | 9,6 | Mannino et al., 2011a |
| Grotta di San Teodoro | Sicily | Upper Palaeolithic (Epigravettian) | -20 | 12,5 | Mannino et al., 2011a |
| Grotta di San Teodoro | Sicily | Upper Palaeolithic (Epigravettian) | -20 | 12 | Mannino et al., 2011a |
| Grotta di San Teodoro | Sicily | Upper Palaeolithic (Epigravettian) | -19,1 | 11,5 | Mannino et al., 2011a |
| Grotta d'Oriente | Sicily | Upper Palaeolithic (Epigravettian) | -19,3 | 11 | Craig et al., 2010 |
| S'Omu e S'Orku | Sardinia | Mesolithic | -19,5 | 9,2 | Floris et al., 2012 |
| S'Omu e S'Orku | Sardinia | Mesolithic | -19,9 | 9,5 | Floris et al., 2012 |
| Fontana Nuova | Sicily | Mesolithic | -19,3 | 11,9 | Di Maida et al., 2019 |
| Fontana Nuova | Sicily | Mesolithic | -19,4 | 12 | Di Maida et al., 2019 |
| Grotta Addaura | Sicily | Mesolithic | -19,3 | 8,7 | Mannino et al., 2011b |
| Grotta Addaura | Sicily | Mesolithic | -19,6 | 9,7 | Mannino et al., 2011b |
| Grotta Molara | Sicily | Mesolithic | -20,2 | 7,1 | Mannino et al., 2011b |
| Grotta Molara | Sicily | Mesolithic | -19,5 | 10,4 | Mannino et al., 2011b |
| Grotta d'Oriente | Sicily | Mesolithic | -17,8 | 10,6 | Mannino et al., 2012 |
| Grotta d'Oriente | Sicily | Mesolithic | -18,9 | 11,3 | Mannino et al., 2012 |
| Grotta dell'Uzzo | Sicily | Mesolithic | -20,2 | 9,7 | Mannino et al., 2015 |
| Grotta dell'Uzzo | Sicily | Mesolithic | -20,5 | 9,4 | Mannino et al., 2015 |
| Grotta dell'Uzzo | Sicily | Mesolithic | -21,1 | 11,35 | Mannino et al., 2015 |
| Grotta dell'Uzzo | Sicily | Mesolithic | -19,5 | 9,75 | Mannino et al., 2015 |
| Grotta dell'Uzzo | Sicily | Mesolithic | -20,3 | 12 | Mannino et al., 2015 |
| Grotta dell'Uzzo | Sicily | Mesolithic | -19,3 | 8,7 | Mannino et al., 2015 |
| Grotta dell'Uzzo | Sicily | Mesolithic | -19,1 | 11,7 | Mannino et al., 2015 |
| Grotta dell'Uzzo | Sicily | Mesolithic | -19,7 | 10,8 | Mannino et al., 2015 |
| Grotta dell'Uzzo | Sicily | Mesolithic | -19,7 | 9,7 | Mannino et al., 2015 |
| Grotta dell'Uzzo | Sicily | Mesolithic | -18,9 | 10,8 | Mannino et al., 2015 |
| Grotta dell'Uzzo | Sicily | Mesolithic | -19,1 | 8,7 | Mannino et al., 2015 |
| Mondeval de Sora | Veneto | Mesolithic | -19,9 | 9,1 | this study; Gazzoni et al., 2011 |

**Table S3.** Relative values of masticatory phases of the three individuals (Riparo Villabruna, Tagliente and Mondeval) and comparative sample of Palaeolithic specimen (Nenderthals, *H. sapiens*).

|  | **phaseII** | **Buccal phase I** | **Lingual phase I** |
| --- | --- | --- | --- |
| Riparo Villabruna | 0,11 | 0,31 | 0.58 |
| Riparo Tagliente | 0,18 | 0,33 | 0,49 |
| Mondeval | 0,26 | 0,27 | 0,47 |
| *H. neanderthalensis* | 0.32 (0.06) | 0.34 (0.07) | 0.34 (0.08) |
| *H. sapiens* | 0.37 (0.08) | 0.19 (0.07) | 0.44 (0.7) |

**Table S4.** Results of Kruskal-Wallis test assessing whether the distribution of masticatory phases of all individuals and *Homo sapiens* only are significantly different between variables (Ecozones; MIS and Geography). Significant p values (<0.05).

| All individuals | KW chi-squared | df | P-value |
| --- | --- | --- | --- |
| PhaseII – Ecozones | 3.09 | 2 | 0,21 |
| PhaseII – MIS | 6.66 | 7 | 0,46 |
| PhaseII – Geography | 7.36 | 7 | 0,39 |
| Buccal – Ecozones | 7,47 | 2 | 0,02 |
| Buccal – MIS | 15.21 | 7 | 0.03 |
| Buccal – Geography | 15.43 | 7 | 0,03 |
| Lingual – Ecozones | 3,31 | 2 | 0,19 |
| Lingual – MIS | 9.13 | 7 | 0.24 |
| Lingual – Geography | 10,44 | 7 | 0,16 |
|  |  |  |  |
| Homo sapiens only |  |  |  |
| PhaseII – Ecozones | 3.09 | 2 | 0,21 |
| PhaseII – MIS | 0,27 | 3 | 0,97 |
| Buccal – Ecozones | 7.5 | 2 | 0,024 |
| Buccal – MIS | 3,46 | 3 | 0,33 |
| Lingual – Ecozones | 6.23 | 1 | 0,0126 |
| Lingual – MIS | 2,943 | 3 | 0,4 |

|  | **DEW** | **MED** |
| --- | --- | --- |
| **MED** | 0.037 |  |
| **SCF** | 1 | 0.23 |

**Table S5.** Mann-Whitney tests of Buccal Phase I after Bonferroni correction.

**Table S6.** MannWhitney U test of MIS-based variable using Buccal phase I.

|  | **2** | **3** | **4** | **5d_a** | **5e** | **5e_5c** | **5e_d** |
| --- | --- | --- | --- | --- | --- | --- | --- |
| **3** | 0,86 |  |  |  |  |  |  |
| **4** | 1 | 1 |  |  |  |  |  |
| **5d_a** | 1 | 1 | 1 |  |  |  |  |
| **5e** | 1 | 1 | 1 | NA |  |  |  |
| **5e_5c** | 1 | 1 | 1 | 1 | 1 |  |  |
| **5e_d** | 1 | 1 | 1 | 1 | 1 | 1 |  |
| **6_5e** | 0,12 | 1 | 1 | 1 | 1 | 1 | 1 |

**Table S7.** Mann-Whitney tests of Buccal Phase I after Bonferroni correction.

|  | Belgium | Croatia | France | Hungary | Israel | Italy | Romania |
| --- | --- | --- | --- | --- | --- | --- | --- |
| Croatia | 1.0000 |  |  |  |  |  |  |
| France | 1.0000 | 1.0000 |  |  |  |  |  |
| Hungary | 1.0000 | 1.0000 | 1.0000 |  |  |  |  |
| Israel | 1.0000 | 0.3444 | 0.4172 | 1.0000 |  |  |  |
| Italy | 1.0000 | 1.0000 | 1.0000 | 1.0000 | 1.0000 |  |  |
| Romania | 1.0000 | 1.0000 | 1.0000 | 1.0000 | 1.0000 | 1.0000 |  |
| Spain | 1.0000 | 1.0000 | 1.0000 | 1.0000 | 1.0000 | 1.0000 | 1.0000 |

**Table S8.** AMOVA analysis. Segregation between *Homo neanderthalensis* and *Homo sapiens.*

|  | **Φst** | **P-value** |
| --- | --- | --- |
| **Geography** | 0,14 | 0,033 |
| **Ecozone** | 0,07 | 0,054 |
| **MIS** | 0,09 | 0,09 |
| **Taxonomy** | 0,28 | <0.001 |

***References***

Binford, L. R. (1981). Bones, ancient men and modern myths. *Academic Press, New York,* 328. ISBN: 0121000354.

Blumenschine, R. J. & Selvaggio, M. M. (1988). Percussion marks on bone surfaces as a new diagnostic of hominid behavior. *Nature.* 333, 763–765.

Blumenschine, R. J. (1995). Percussion marks, tooth marks, and experimental determinations of the timing of hominid and carnivore ace to long bones at FLK Zinjanthropus, Olduvai Gorge,Tanzania. *Journal of human Evolution,* 27, 197–213.

Bökönyi, S. (1970). A new method for determination of the number of individuals in animal bone material. *American Journal of Archaeology,* 74, 291–292.

Brain, C. K. (1981). The hunters or the hunted? An introduction to African Cave Taphonomy. *University of Chicago Press, Chicago,* 376. ISBN: 9780226070902.

Capaldo, S. D. & Blumenschine, R. J. (1994). A quantitative diagnosis of notches made by hammerstone percussion and carnivore gnawing on bovid long bones. *American Antiquity,* 59, 724–748.

D’Errico, F., Vanhaeren, M. (2002). Criteria for identifying red deer (Cervus elaphus) age and sex from their canines: application to the study of Upper Palaeolithic and Mesolithic ornaments. *Journal of Archaeological Science,* 29, 211-232.

Von Den Driesch, A. (1976). Guide to the Measurement of Animal Bones from Archaeological Sites. *MA: Peabody Museum Bulletin 1*, Cambridge.

Fisher, W. J. (1995). Bone surface modifications in zooarchaeology. *Journal of* Archaeological Method *and Theory,* 2, 7–68.

Fiore I. & Tagliacozzo A., 2006 - Lo sfruttamento dello stambecco nel Tardiglaciale di Riparo Dalmeri (TN): il livello 26c. In Tecchiati U. & Sala B. (a cura di), Archaeozoological studies in honour of Alfredo Riedel. Ufficio Beni Archeologici, Bolzano: 59-76.

Gazzoni, V., Goude, G., Herrscher, E. Guerreschi, A., Antonioli, F. & Fontana, F. (2013). Late Upper Palaeolithic human diet: first stable isotope evidence from Riparo Tagliente (Verona, Italy). *Bulletins et mémoires de la Société d'anthropologie de Paris* 25, 103-117.

Grayson, D.K. (1984). Quantitative Zooarchaeology: Topics in the Analysis of Archaeological Faunas. *Academic Press*, Orlando, 202.

Kromer, B., Lindauer, S., Synal, H.-A., & Wacker, L. (2013). MAMS – A new AMS facility at the Curt-Engelhorn-Centre for Achaeometry, Mannheim, Germany. *Nuclear Instruments and Methods in Physics Research section B,* 294, 11-13.

Mariezkurrena, K. (1983). Contribution al conocimiento del desarrollo de la denticion y el esqueleto postcraneal de Cervus elaphus*. Munibe.* 35, 149-202.

Potts, R. & Shipman, P. (1981). Cutmarks made by stone tools on bones from Olduvai Gorge, Tanzania. *Nature*. **291**, 577–580.

Shipman, P. (1981). Life history a fossil. An introduction to taphonomy and paleoecology. *Harvard University Press, Harvard*, 222.

Shipman, P. & Rose, J. (1984). Cutmark mimics on modern fossil bovid bones. *Current Anthropology*, 25, 116–177.

Stiner, M.C., Kuhn, S.L., Weiner, S., & Bar-Yosef, O. (1995). Differential Burning, Recrystallization and Fragmentation of Archaeological Bone. *Journal of Archaeological Science,* 22, 223-237.

Van Klinken, G. J. (1999). Bone Collagen Quality Indicators for Palaeodietary and Radiocarbon Measurements. *Journal of Archaeological Science,* 26, 687-695.

Vigal, C.R., & Machordom, A. (1985). Tooth eruption and replacement in the Spanish wild goat. *Acta theriologica*, 30, 305-320.

Wick, L. (1994). Early-Holocene reforestation e vegetation change at a lake near the Alpine forest limit: Lago Basso (2250 m asl), Northern Italy. Lotter, A. F. & Ammann, B. (ed.), *Dissertationes Botanicae*, 555-563.
